# Supplementary material for: Combining Genetic and Transcriptomic Approaches to Identify Transporter-Coding Genes as Likely Responsible for a Repeatable Salt Tolerance QTL in Citrus
Source: Int J Mol Sci. 2023 Oct 30;24(21):15759. doi: 10.3390/ijms242115759 (PMC10650496; doi:10.3390/ijms242115759)
Supplement: Supplementary file 1 [file ijms-24-15759-s001.zip › ijms-2636745-supplementary.pdf]

International Journal of *Molecular Sciences*

Article

Combining Genetic and Transcriptomic Approaches to Identify Transporter-Coding Genes as Likely Responsible for a Repeatable Salt Tolerance QTL in Citrus

Figure S1. General views of the GP experiment (A, 2014; B, 2013) at the Valencia facility and the NG experiment (C, 2020) at the Malaga facility, both in Spain.

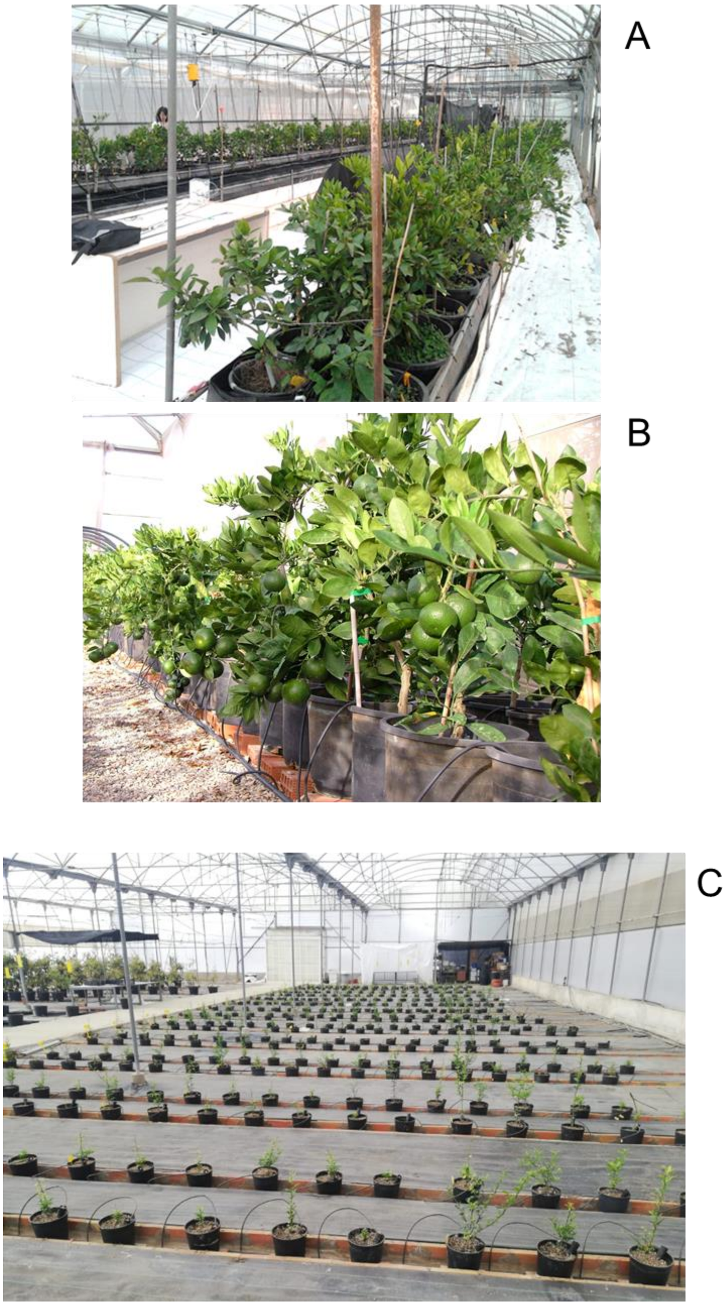

Figure S2. Distributions of relevant traits evaluated in GP experiment under control (green) and salinity (red) conditions. The means of the parents (Cleopatra and Flying Dragon) and the reference rootstock Carrizo are indicated by black, red and blue vertical lines, respectively. Discontinuous lines correspond to control means.

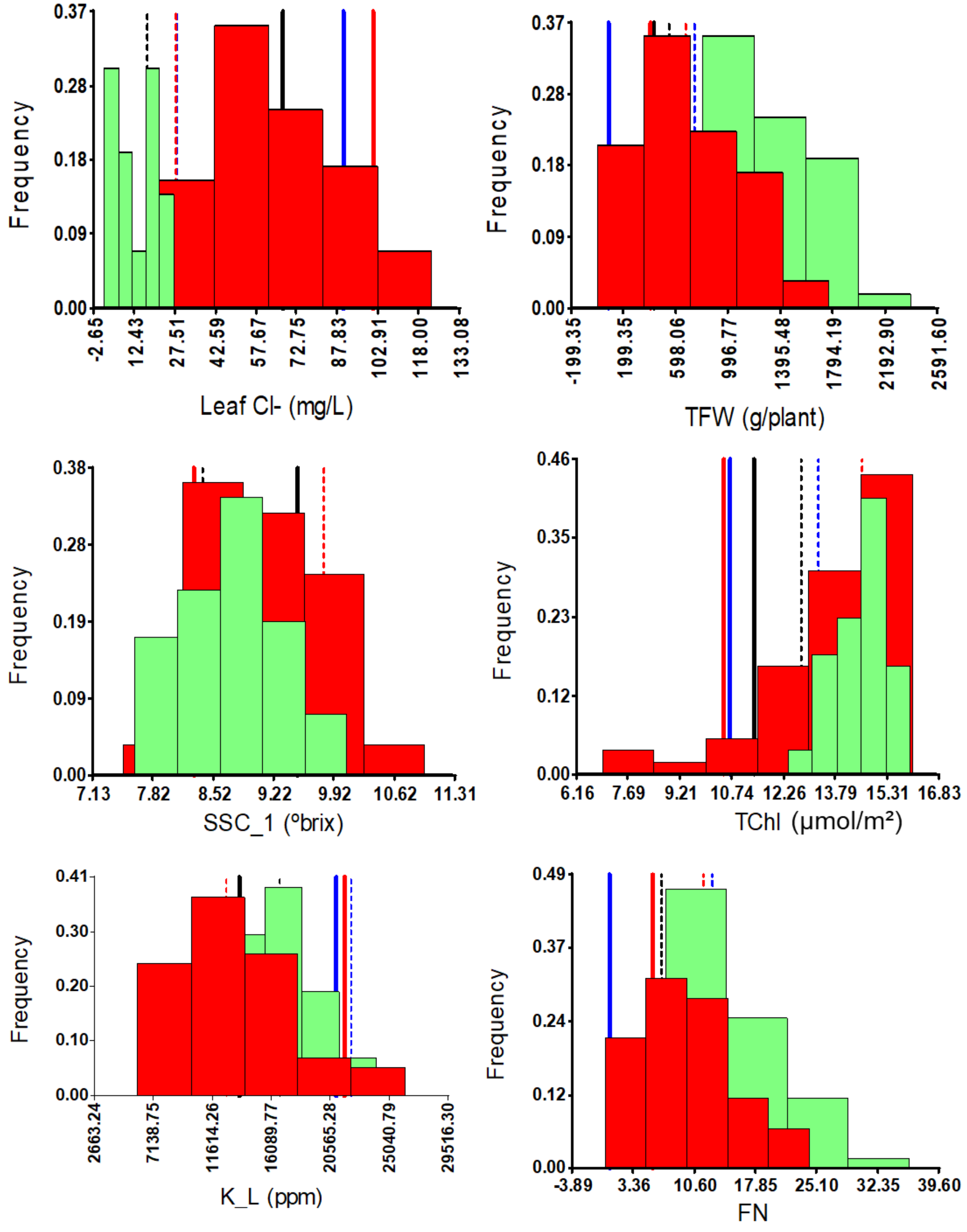

Figure S3. A: LOD profiles of QTLs in linkage group 4c (*C. clementina* scaffold 6), including one involved in rootstock-mediated tolerance to salinity (Cl_L_S) in GP experiment (Cl*_S corresponds to the salt tolerance QTL reported by Raga *et al.*, 2016). Genetic positions (markers) along the integrated *Citrus-Poncirus* linkage group 4c are shown under the X axis. Linkage group- and genome- wide LOD critical values are indicated by a thin and a thick horizontal red line, respectively. B: Genotypic means at significant marker CR23,750.

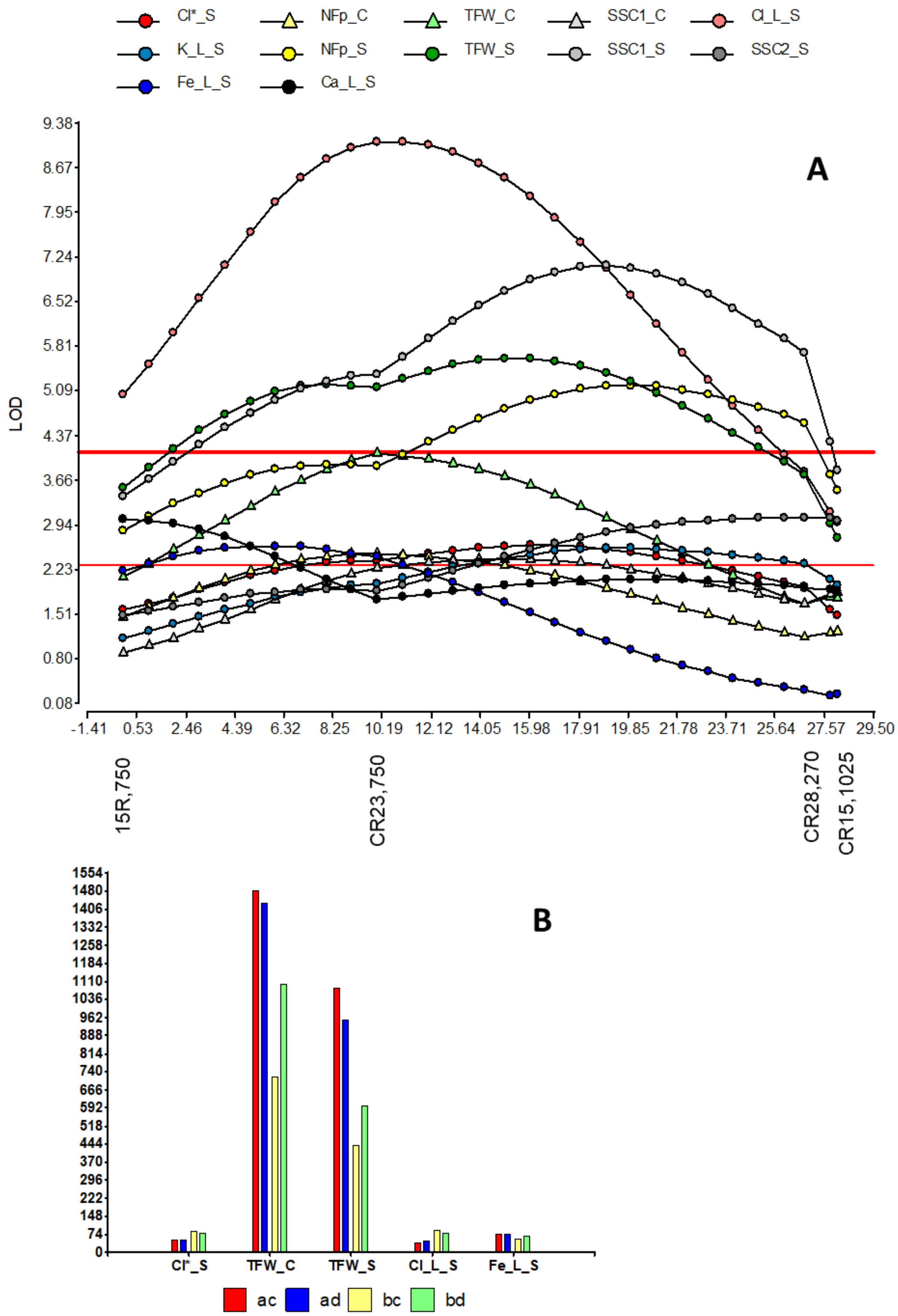

Figure S4. Distributions of traits related to leaf and root [Cl$^-$] in the NG experiment, under control conditions, in green, and, under salinity conditions, in red: (A) leaf [Cl$^-$], (B) root [Cl$^-$], and (C) their difference relative to root [Cl$^-$]; the means of the parents (Cleopatra, in black; Rich, in red) are indicated by vertical lines. Discontinuous lines correspond to control means.

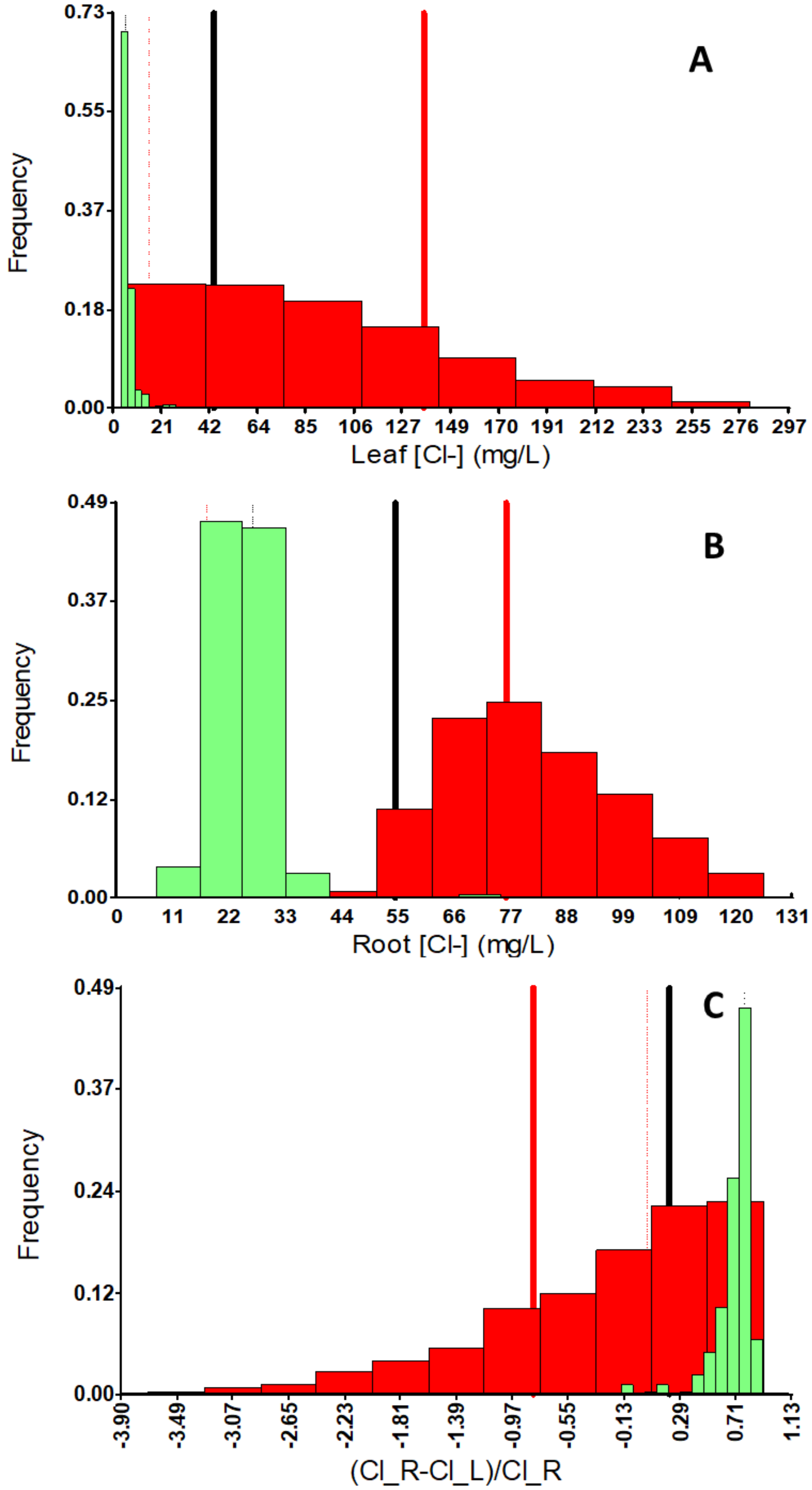

Figure S5. Reaction norm plots of hybrids and parents (salinity responses) for leaf [Cl^-] (A), root [Cl^-] (B), their difference relative to root [Cl^-] (C), total root dry weight (E), and fine root dry weight (D). Robustness (small slope) in A and C is related to salt tolerance. G×E interactions were significant in traits depicted in A, B, C, and E (Table S3). G×E interactions in A, B, and C are driven by changes in variances between salinity levels, while those in E are driven by changes in ranking of responses to salinity.

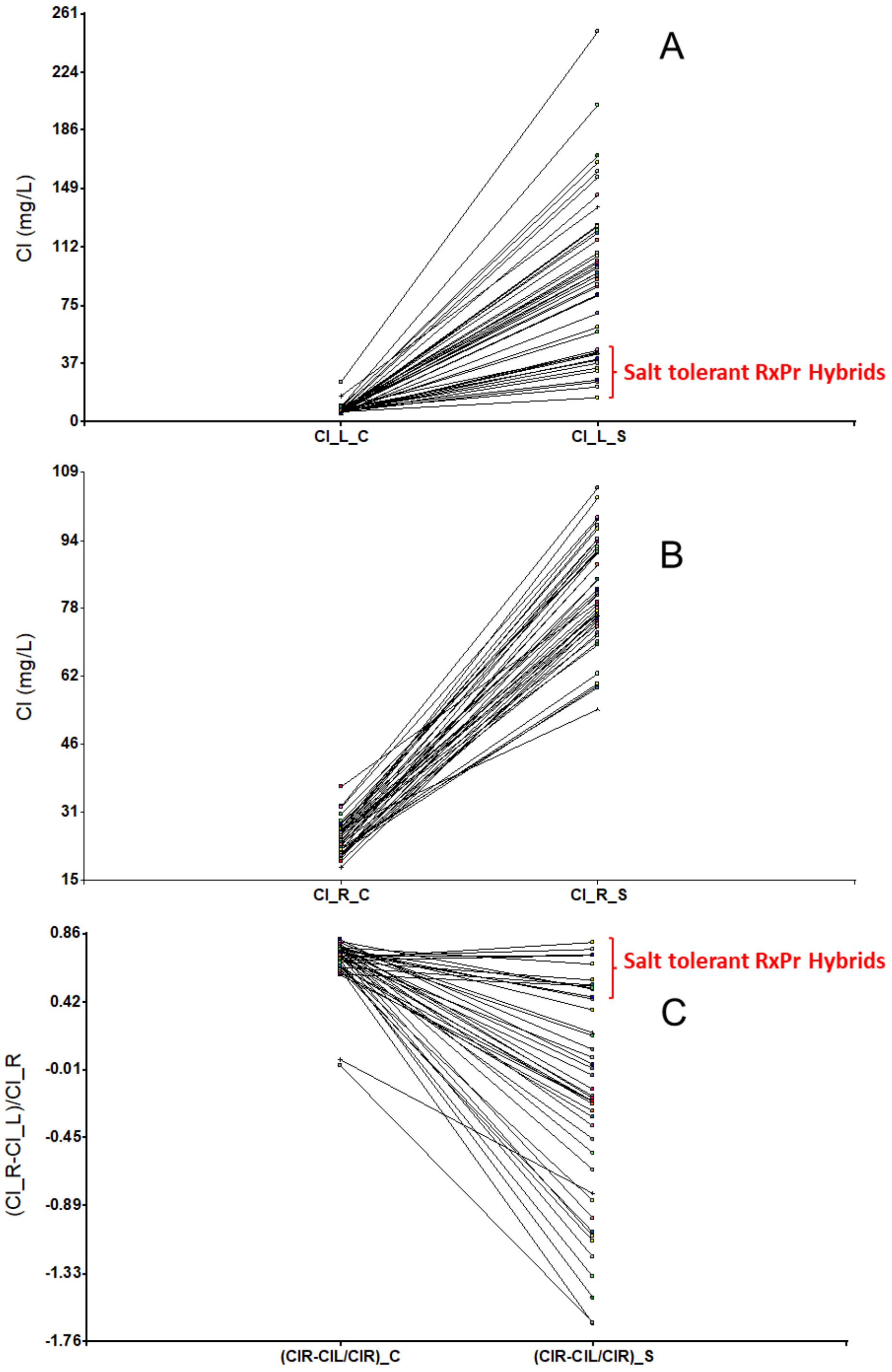

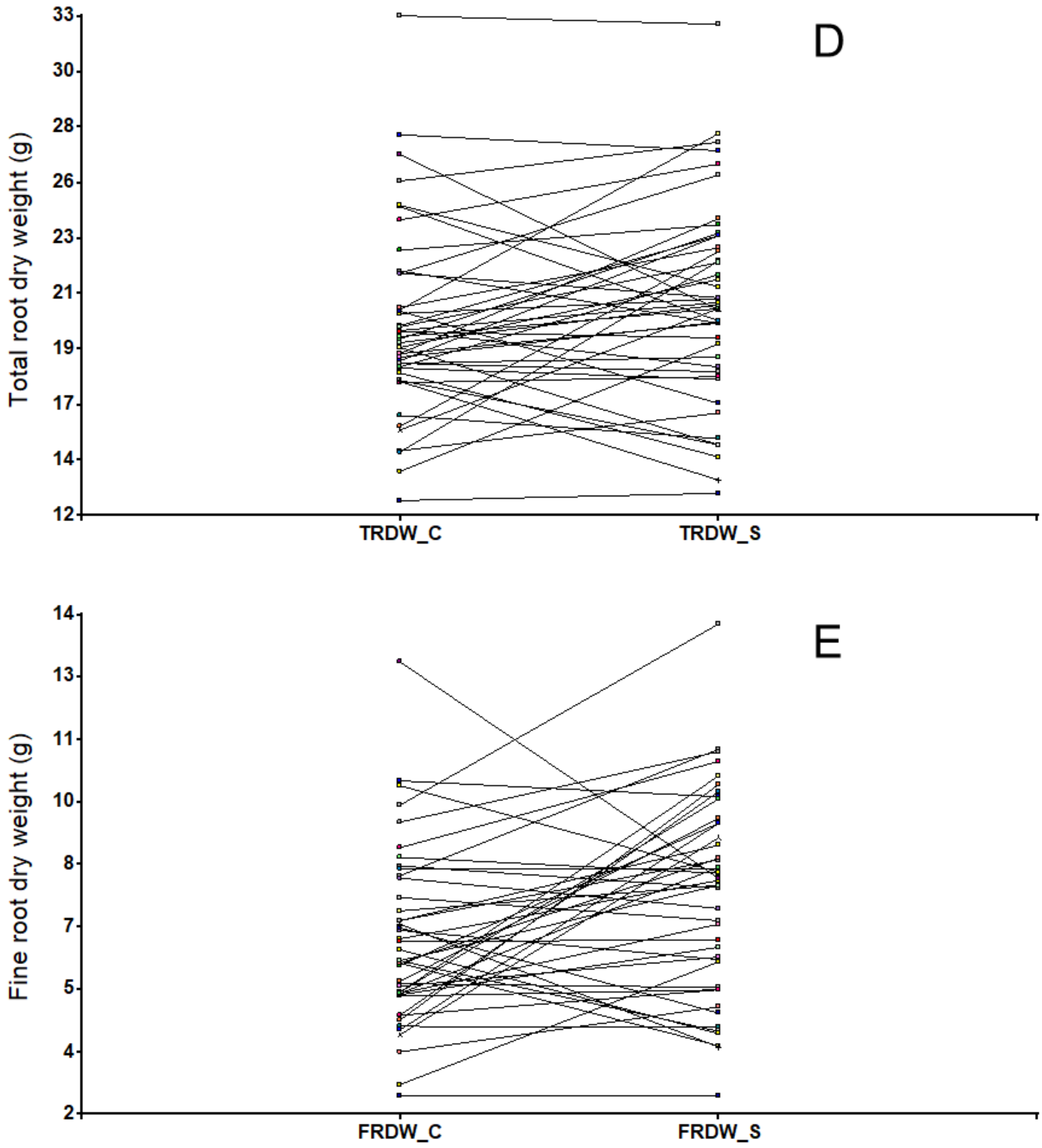
D
Total root dry weight (g)
TRDW_C
TRDW_S
E
Fine root dry weight (g)
FRDW_C
FRDW_S

Figure S6. Means and standard deviations for total root dry weight (TRDW) and fine root dry weight (FRDW) of hybrids and parents (Cleopatra, Cleo, and trifoliata orange, Rich). Salt-tolerant and salt-sensitive hybrids (107 and 90, respectively) are indicated by an arrow (black and red, respectively).

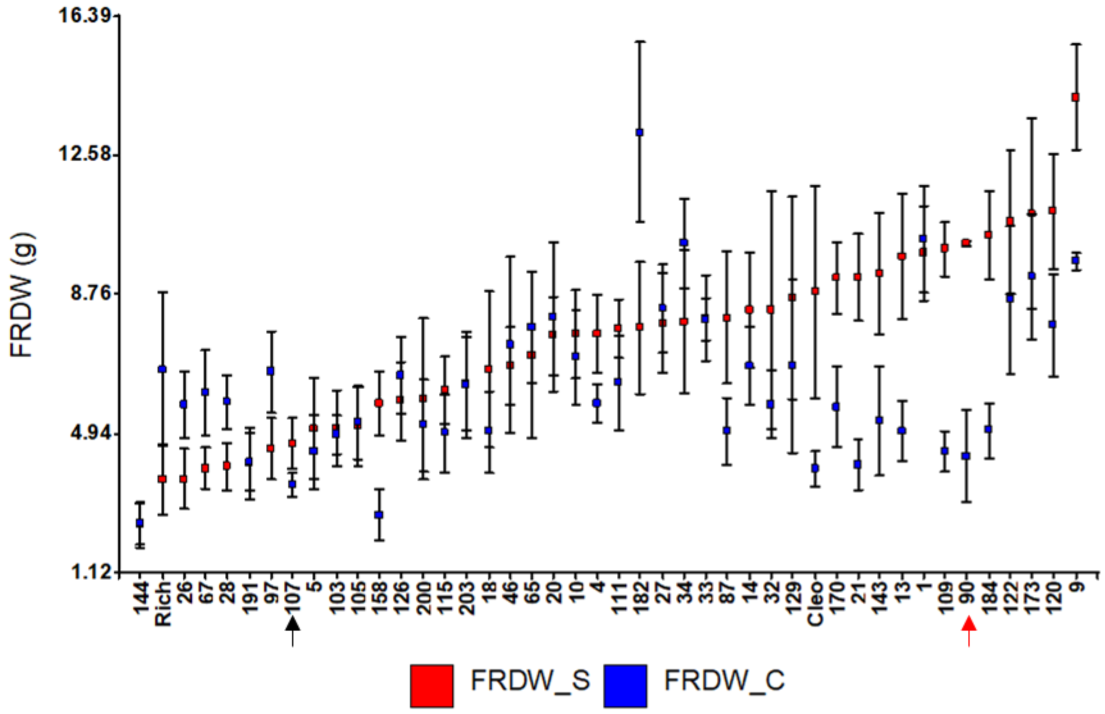

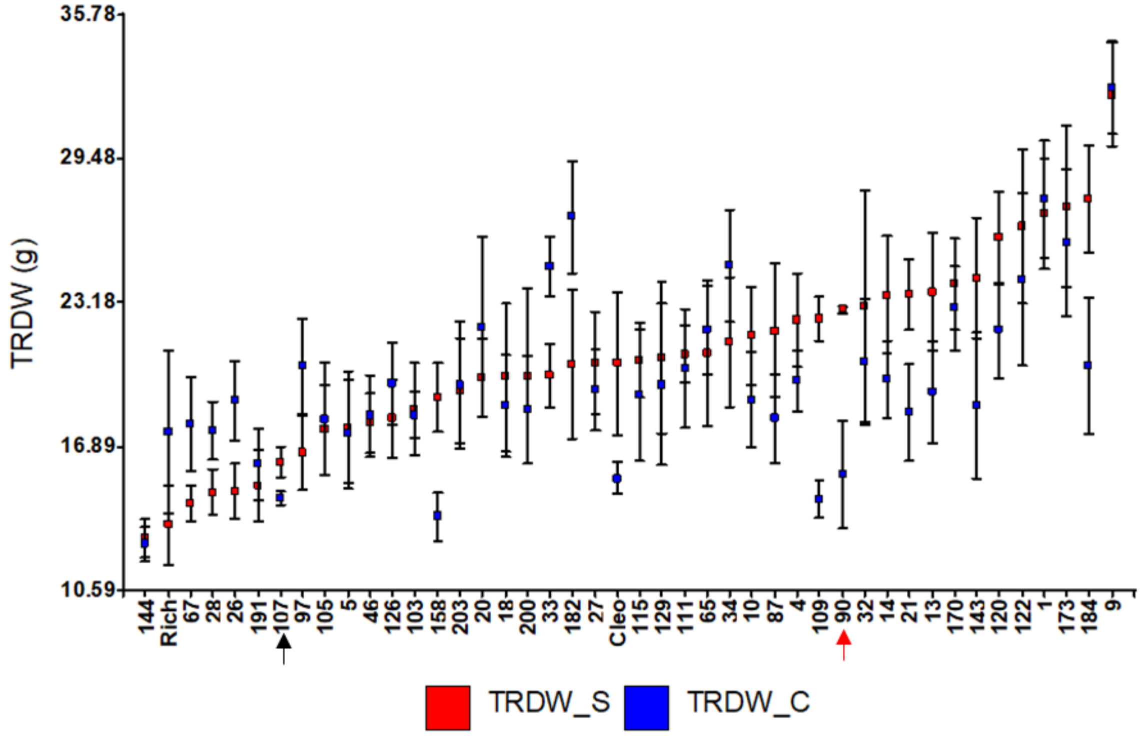

Figure S7. LOD profiles of QTLs in linkage group 4c (*C. clementina* scaffold 6), including those related to leaf Cl- under salinity conditions (Cl_L_S, dCl_ and (ClR-ClL/ClR)_S) in NG experiment. Genetic positions (markers) along the integrated *Citrus reshni-Poncirus trifoliata* linkage group 4c are shown under the X axis. Linkage group- and genome-wide LOD critical values are indicated by a thin and a thick horizontal red line, respectively.

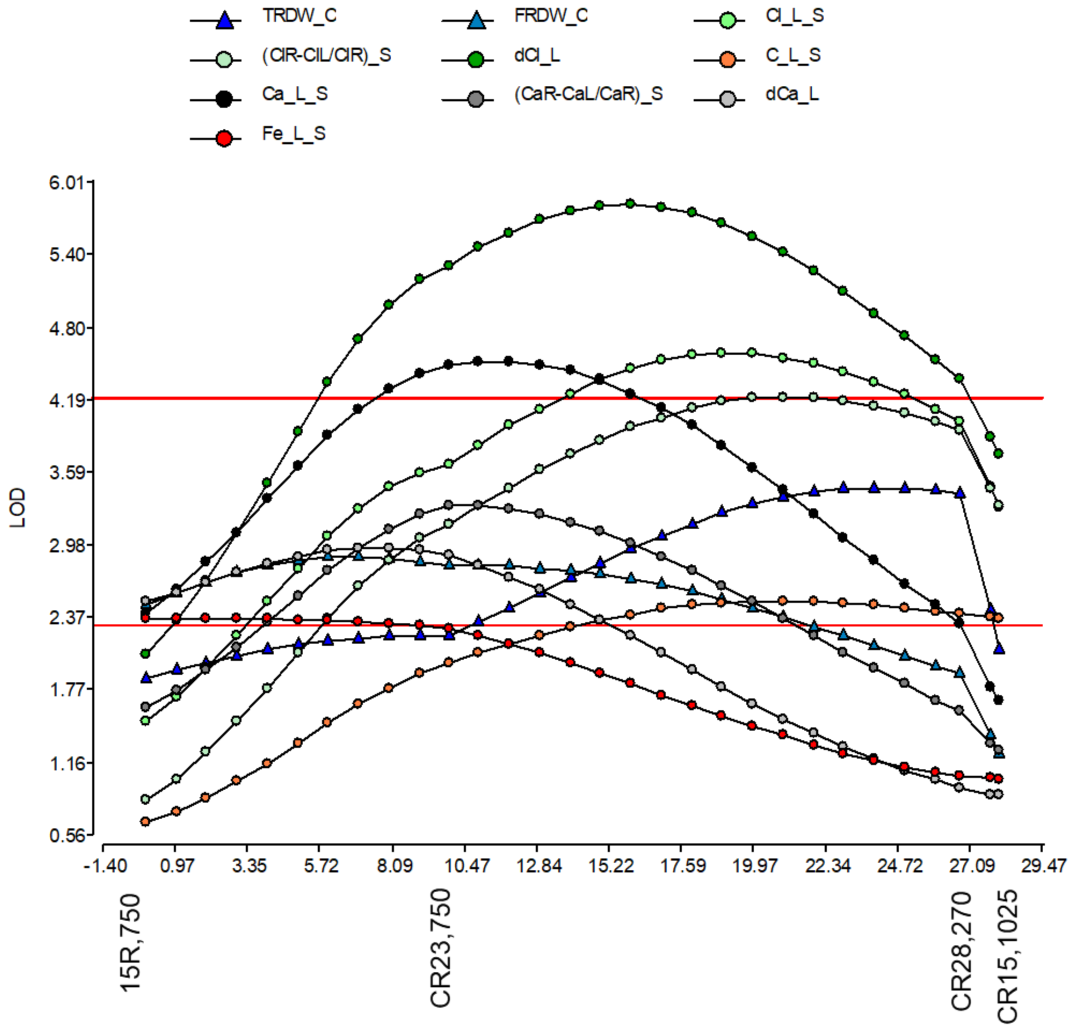

Figure S8. Genotypic means at significant marker CR23,750 for Cl_L_S (divided by 10), Ca_L_S (divided by 1000), dCl_L, (ClR-ClL/ClR)_S, (CaR-CaL/CaR)_S and dCa_L.

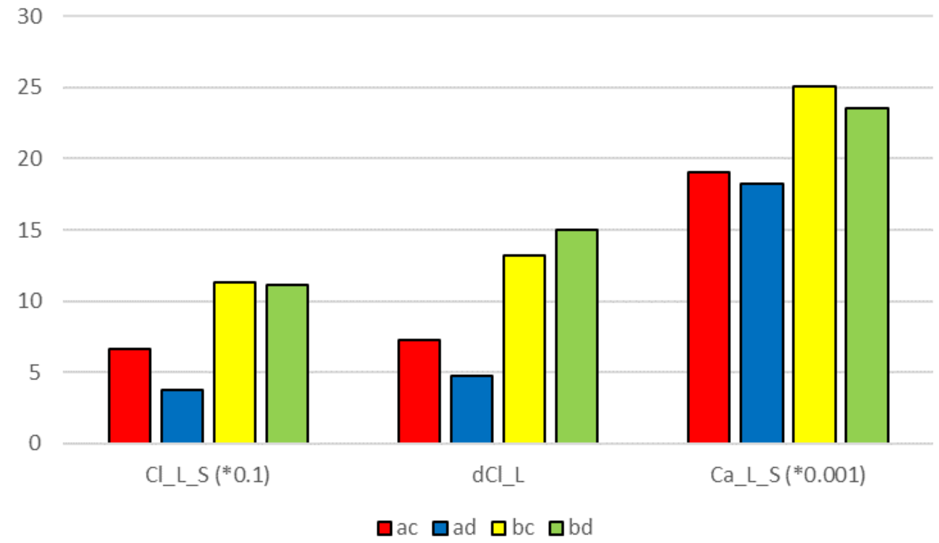

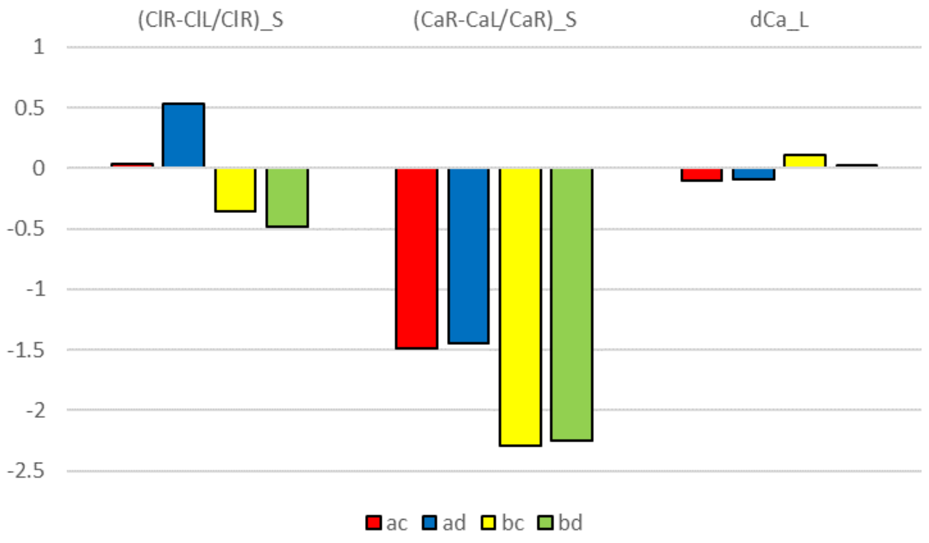

Figure S9. Alignment of translated sequences for alleles at locus NPF5.9 (Ciclev10013337m/Potrif.0006s0823.1) in 22-7 (bb), 107 (ac) and 90 (bc) trees.

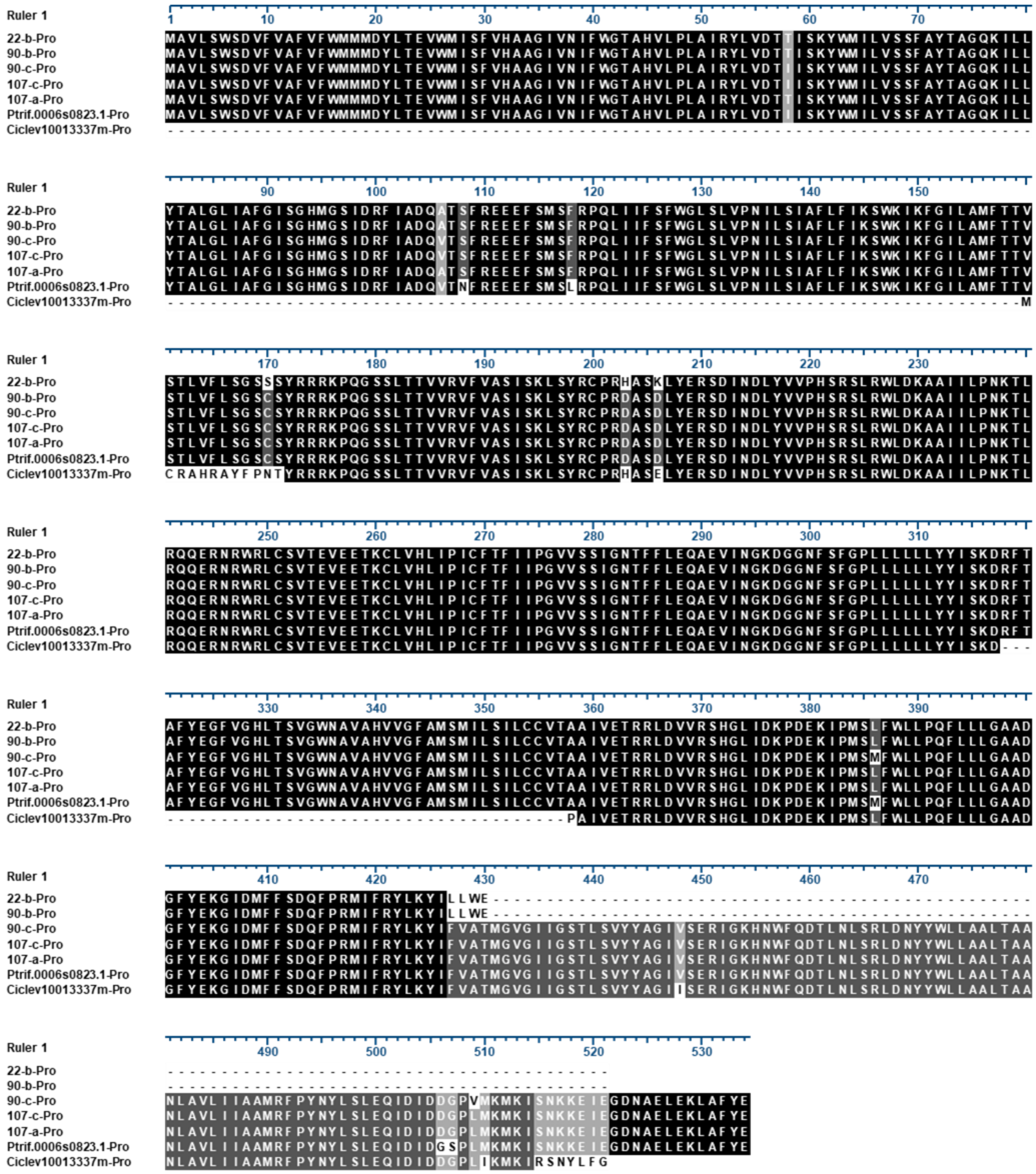

Figure S10. Alignment of translated sequences for alleles at locus PIP2.1 (Ciclev10012375m/Ptrif.0006s0419.1) in 22-7 (bb), 107 (ac) and 90 (bc) trees.

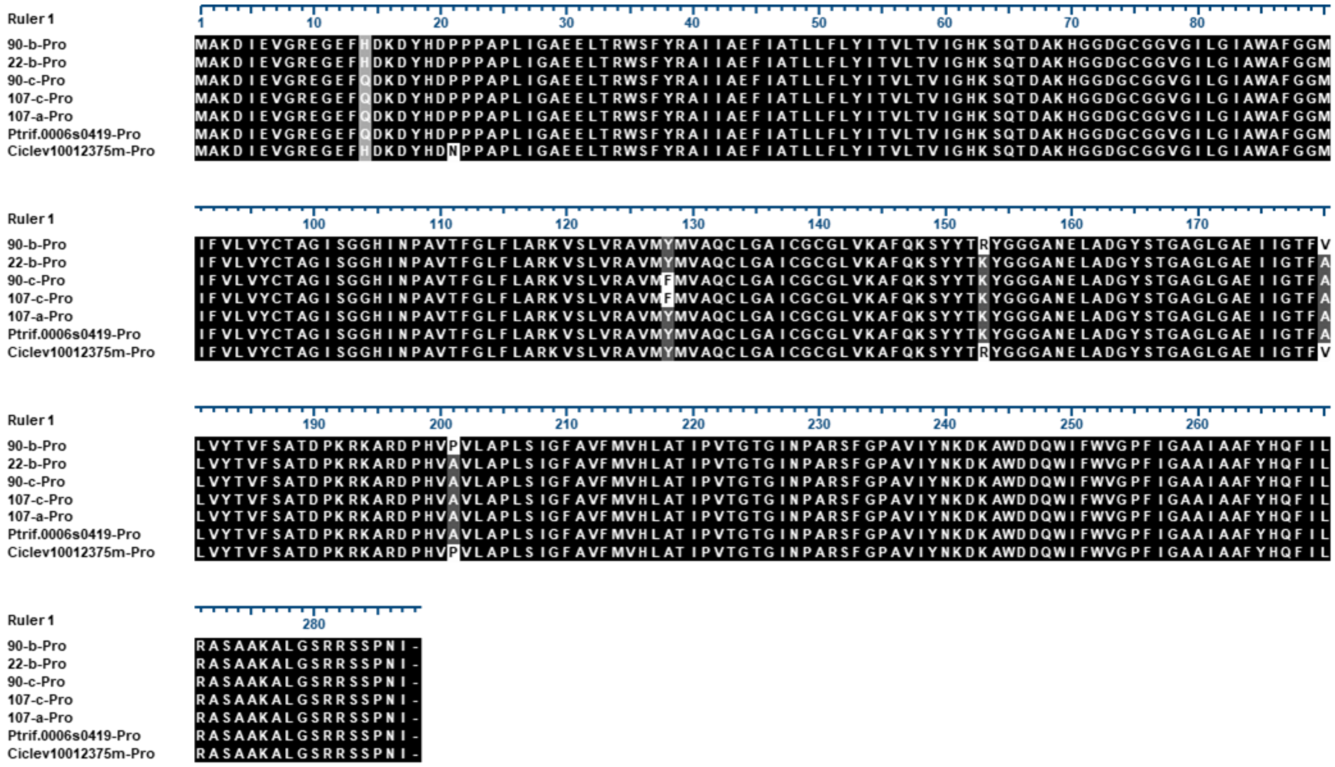

Figure S11. Alignment of translated sequences for alleles at Major Facilitator Superfamily protein locus Ciclev10011745m/Ptrif.0006s0610.1 in 22-7 (bb), 107 (ac) and 90 (bc) trees.

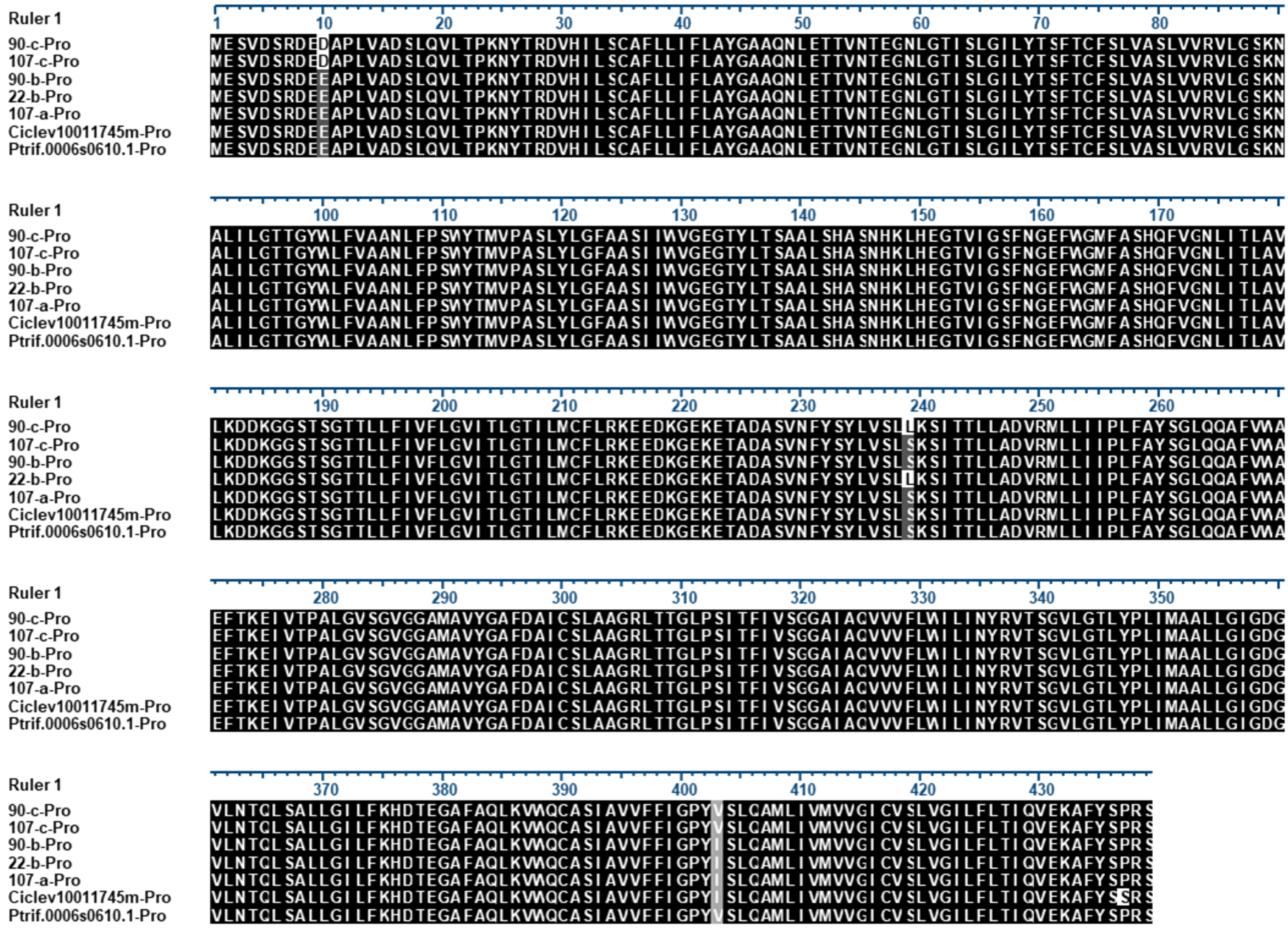

Figure S12. Read mapping of candidate genes coding for NPF5.9 (A), MSF (B), NPF5.12 (C), NPF8.2 (D) and PIP2.1 (E) using the genomic sequences (alleles a and b from *C. reshni)* as reference. In all cases, the upper mapping corresponds to reads from genotype 107, while the bottom mapping corresponds to reads from genotype 90; the range in the top left part (below the protein and allele) reflects coverage as a surrogate of expression level. The red lines and names correspond to the predicted transcript of *C. clementina* that codes for the corresponding protein. In the case of MSF (B), the green and blue lines and names correspond to alternative isoforms described for the same gene. Black/gray lines and names for NPF5.9 (A) and NPF8.2 (D) correspond to orthologous transcripts that explain the mapping in this region. In panel A, XM_006481338 corresponds to a protein similar to that belonging to the NRT1/PTR family 5.5 (NPF5.5) of proton-dependent nitrate and oligopeptide transporters (DOI: 10.1038/srep07962), XM_024181054 is a predicted *Citrus clementina* putative disease resistance protein RGA3, XM_052437161 is a *Citrus sinensis* uncharacterized protein, and XM_025095325 is also a *Citrus sinensis* hypothetical protein. In panel D, the transcribed regions surrounding ESR42548 did not match any significant predicted orthologue in plants.

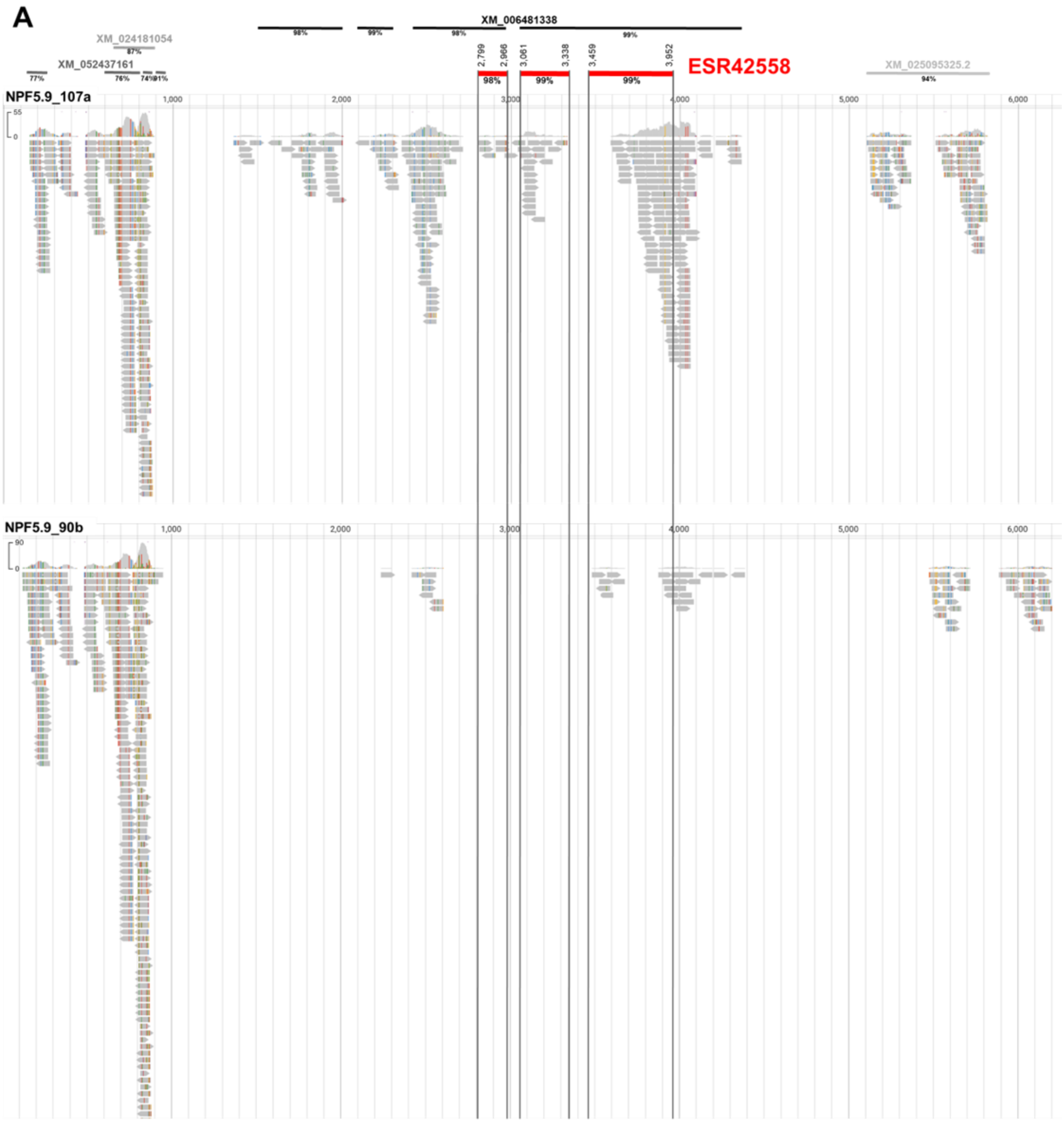
A
XM_024181054
87%
XM_052437161
77%
76%
74%
91%
98%
99%
98%
XM_006481338
99%
2,799
2,966
3,061
3,338
3,459
3,952
ESR42558
98%
99%
99%
XM_025095325.2
94%
NPF5.9_107a
1,000
2,000
3,000
4,000
5,000
6,000
55
0
NPF5.9_90b
90
0

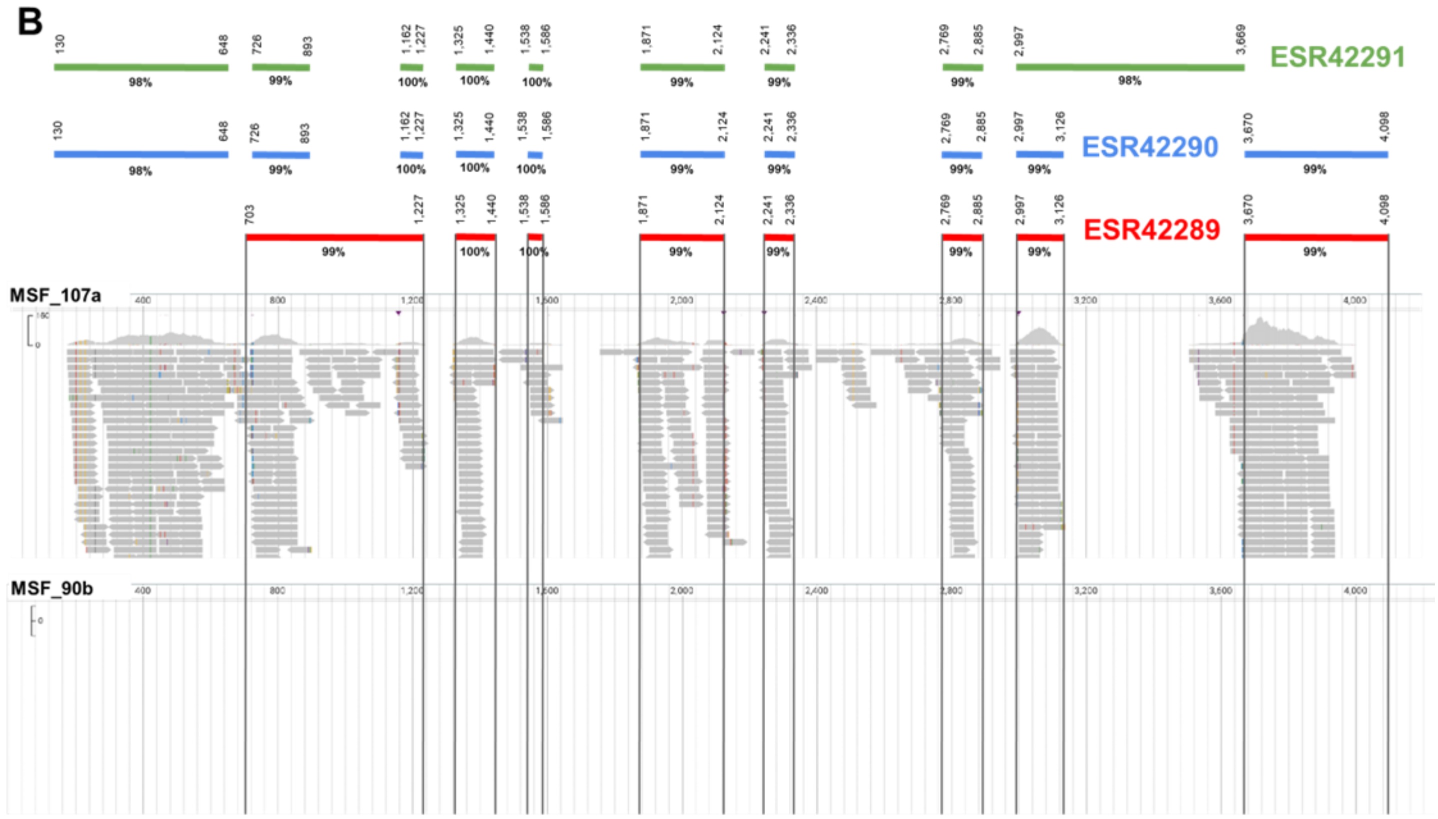
B
ESR42291
ESR42290
ESR42289
MSF_107a
MSF_90b

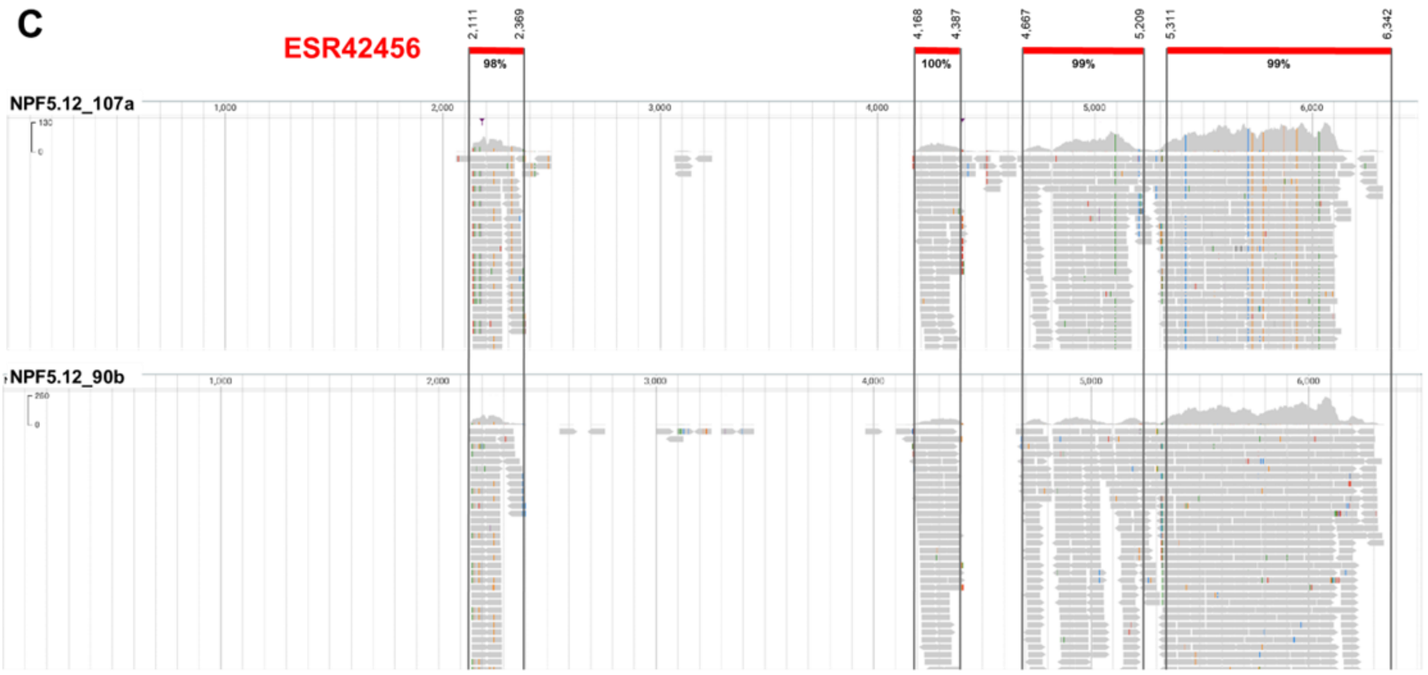
C
ESR42456
NPF5.12_107a
NPF5.12_90b

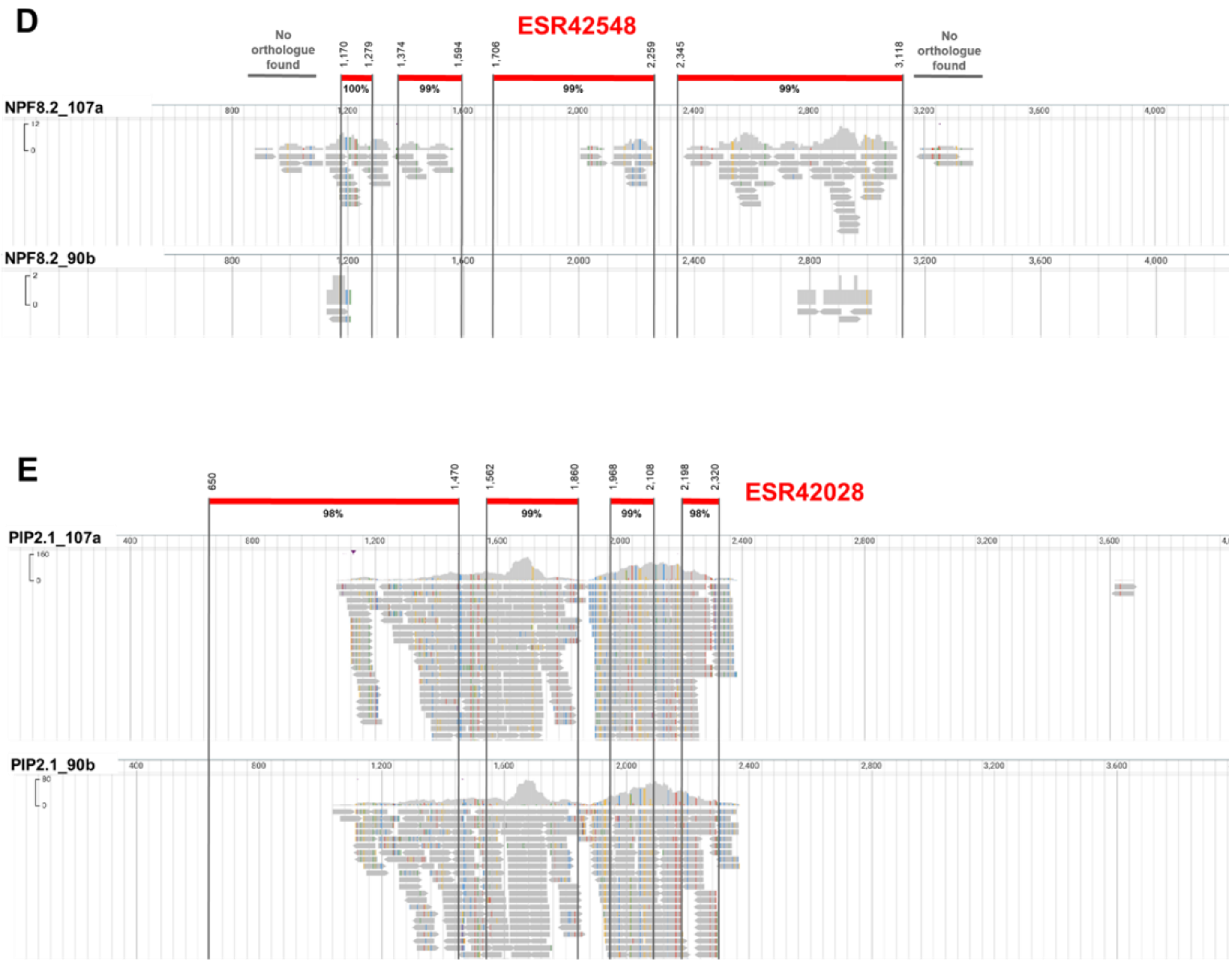
D
ESR42548
No orthologue found
1,170
1,279
1,374
1,594
1,706
2,259
2,345
3,118
No orthologue found
100%
99%
99%
99%
NPF8.2_107a
NPF8.2_90b
800
1,200
1,600
2,000
2,400
2,800
3,200
3,600
4,000
E
650
1,470
1,562
1,860
1,968
2,108
2,198
2,320
ESR42028
98%
99%
99%
98%
PIP2.1_107a
PIP2.1_90b
400
800
1,200
1,600
2,000
2,400
2,800
3,200
3,600

Figure S13. Expression patterns represented as normalized mean counts per million (cpm) reads mapped after agglomerative hierarchical clustering (AHC) for DEGs in *LCl-6*. Cpm was normalized using the CTF method and then scaled, the similarity matrix was obtained using Euclidean distances, Ward's minimum variance method was used for linkage, and, finally, a dynamic clustering approach was used to group genes in clusters AHC-1, AHC-2 and AHC-3. It can be seen that genes in AHC-1 exhibit a slight increase in expression under salt stress conditions in 107, but decreased in 90.

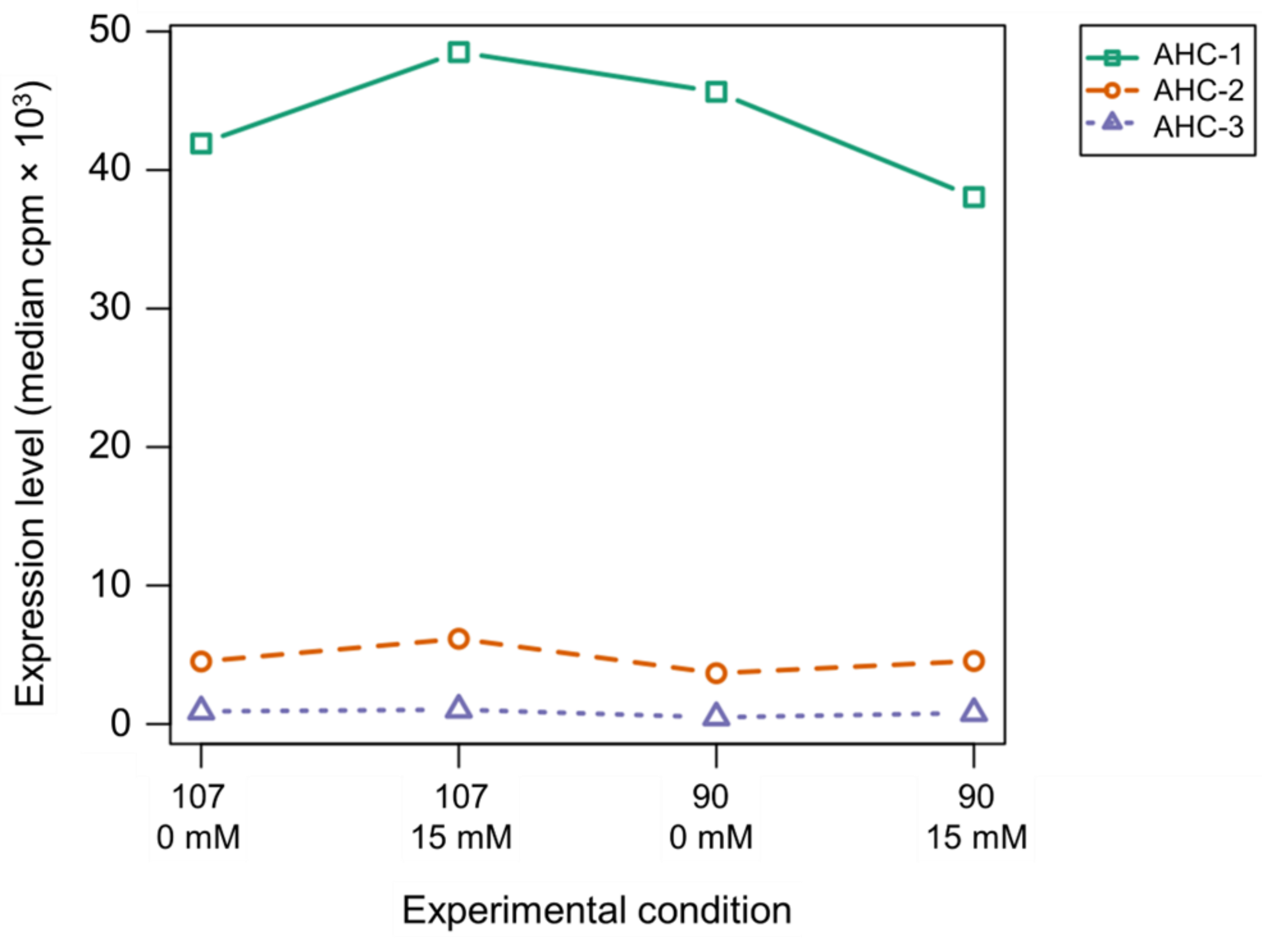

Figure S14. Relationship between genetic (X axis) and physical (Y axis) positions for linkage groups 4c (A) and R4c (B) in *C. clementina* scaffold 6.

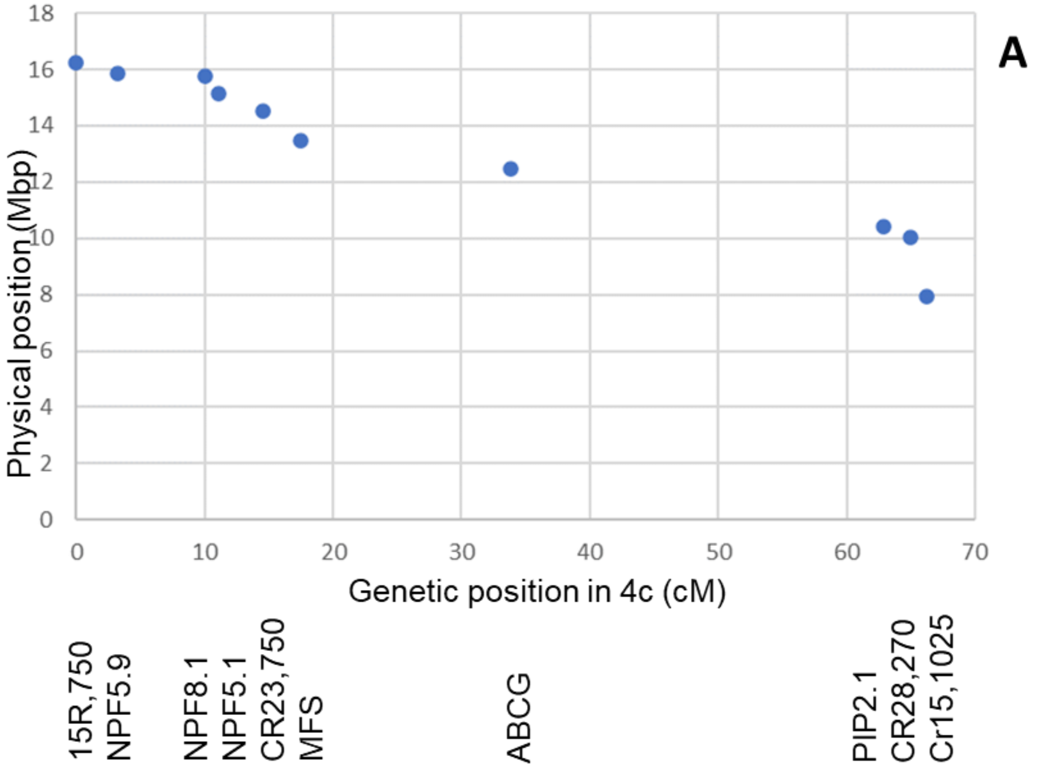

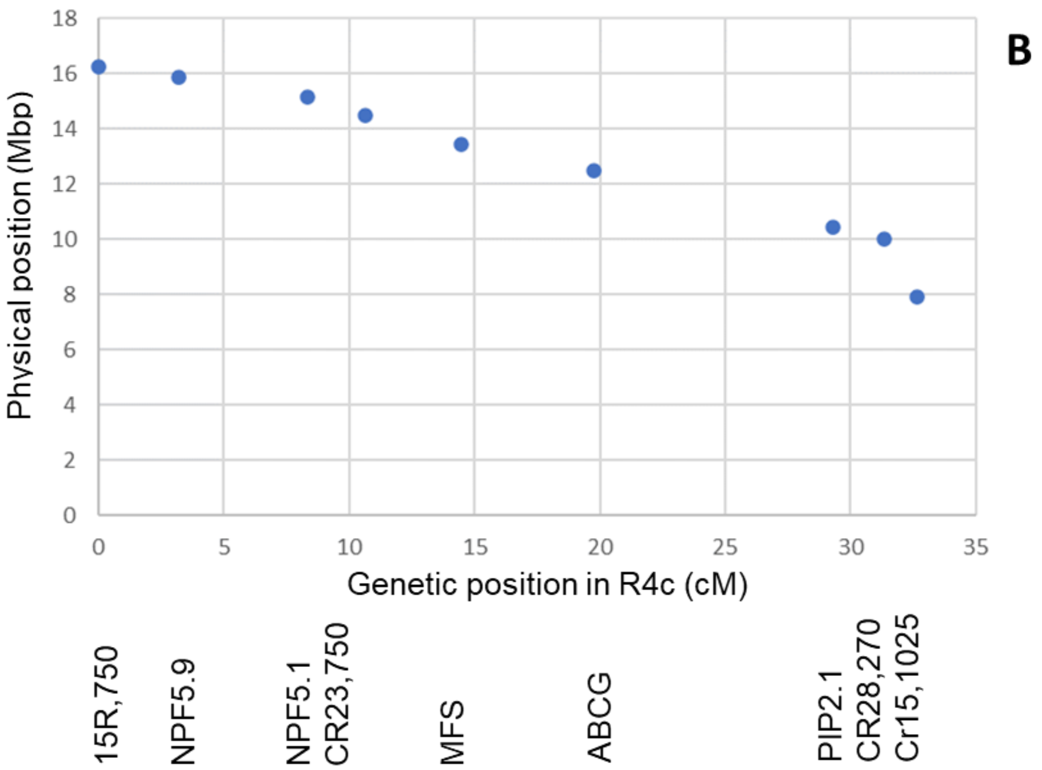

Figure S15. LOD profiles of QTLs for fruit yield- (NFp and TFW) and root mass- (TRDW and FRDW) related traits and leaf Fe under salinity conditions in the grafted-population experiment. The X axis shows linkage map group 4c after incorporating candidate genes. The red horizontal line indicates significant level.

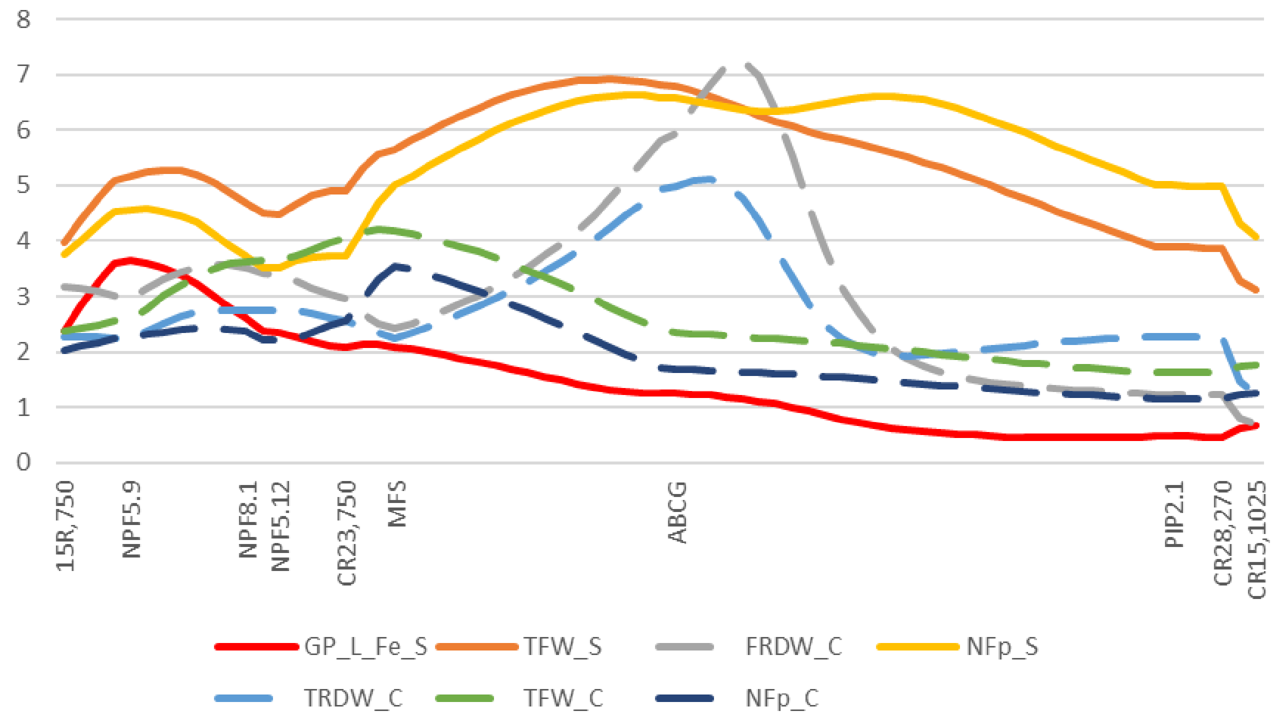

International Journal of *Molecular Sciences*

MDPI

Table S1. Trait abbreviations and heritability estimates (H2_C and H2_S for control and salinity treatments, respectively) and *p* values for the significant effects in the mixed model analysis of evaluated traits in GP experiment. G and E denote genotype and salinity effects, respectively. ns: non-significant, ne: not estimated.

Trait Abbreviation	Trait	H2_C	H2_S	G	E	GxE
A1	Juice titrable acidity at 1st harvesting date	0.0669	0.2001	ns	ns	ne
A2	Juice titrable acidity at 2nd harvesting date	0.0510	0.0289	ns	ns	ne
Ca_L	Leaf Ca concentration (ppm)	0.0000	0.2326	0.0099	ns	ns
Cl_L	Leaf Cl concentration (mg L^{-1})	0.2397	0.4027	<0.0001	ns	<0.0001
Fe_L	Leaf Fe concentration (ppm)	0.0731	0.2525	0.0005	ns	ns
FW1	Fruit weight 1st harvesting date (g)	0.0695	0.0071	ns	ns	ne
FW2	Fruit weight 2nd harvesting date (g)	0.0000	0.0831	ns	0.0226	ne
K_L	Leaf K concentration (ppm)	0.0000	0.1247	0.01	0.0137	ns
Na_L	Leaf Na concentration (ppm)	0.1153	0.2576	<0.0001	0.0003	0.0001
NFp	number of normal, ripe fruits	0.1582	0.1790	ne	ne	ne
SSC1	Juice soluble-solids content at 1st harvesting date	0.1198	0.3490	<0.0001	0.037	ne
SSC2	Juice soluble-solids content at 2nd harvesting date	0.0248	0.4587	<0.0001	ns	ne
TChl	total chlorophyll leaf concentration (μmol m^{-2})	0.0000	0.1015	ns	0.0376	0.0223
TFW	total fruit weight (g)	0.2601	0.3097	<0.0001	0.0048	ns

Table S2. Spearman coefficients (r) of significantly correlated traits (*p*<0.05) among traits evaluated in the GP experiment under control and salinity conditions, and for each trait under both salinity conditions.

Trait 1_C	Trait 2_C	r	*p*-value
A1	A2	0.57	<0.0001
A1	NFp	0.38	0.0050
A1	SSC1	0.67	<0.0001
A1	SSC2	0.61	<0.0001
A1	TFW	0.28	0.0411
A2	SSC2	0.44	0.0012
Ca_L	A2	-0.42	0.0020
Ca_L	Cl_L	0.54	<0.0001
Ca_L	K_L	-0.39	0.0035
Ca_L	Na_L	0.57	<0.0001
Cl_L	A2	-0.33	0.0165
Cl_L	K_L	-0.30	0.0243
Cl_L	Na_L	0.88	<0.0001
Fe_L	NFp	0.30	0.0257
Fe_L	TFW	0.39	0.0032
FW1	A1	-0.52	0.0001
FW1	K_L	0.29	0.0370
FW1	NFp	-0.37	0.0068
FW1	SSC1	-0.46	0.0005
FW1	SSC2	-0.37	0.0071
Na_L	A2	-0.41	0.0023
NFp	SSC1	0.54	<0.0001
NFp	SSC2	0.47	0.0005
NFp	TFW	0.95	<0.0001

Trait 1_S	Trait 2_S	r	*p*-value
A1	A2	0.60	<0.0001
A1	SSC1	0.38	0.0048
A1	SSC2	0.51	0.0001
A1	TChl	-0.40	0.0031
A2	SSC2	0.30	0.0323
A2	TChl	-0.41	0.0022
Ca_L	Fe_L	0.64	<0.0001
Ca_L	K_L	-0.62	<0.0001
Ca_L	NFp	0.47	0.0002
Ca_L	TChl	0.29	0.0313
Ca_L	TFW	0.41	0.0019
Cl_L	Fe_L	-0.43	0.0015
Cl_L	K_L	0.31	0.0198
Cl_L	Na_L	0.57	<0.0001
Cl_L	NFp	-0.46	0.0003
Cl_L	SSC1	-0.48	0.0003
Cl_L	SSC2	-0.39	0.0047
Cl_L	TChl	-0.50	0.0002
Cl_L	TFW	-0.59	<0.0001
Fe_L	K_L	-0.39	0.0038
Fe_L	NFp	0.32	0.0137
Fe_L	TChl	0.49	0.0002
Fe_L	TFW	0.40	0.0031
FW1	A1	-0.45	0.0006

Trait_C	Trait _S	r	*p*-value
Na_L_C	Na_L_S	-0.38	0.0049
NFp_C	NFp_S	0.28	0.0372
SSC_1_C	SSC_1_S	0.39	0.0043
TFW_C	TFW_S	0.39	0.0034

SSC1	SSC2	0.66	<0.0001
SSC1	TFW	0.52	0.0001
SSC2	TFW	0.37	0.0070

FW1	A2	-0.28	0.0428
FW1	K_L	0.28	0.0418
FW1	NFp	-0.32	0.0178
FW1	SSC1	-0.49	0.0002
FW1	SSC2	-0.45	0.0008
K_L	NFp	-0.60	<0.0001
K_L	SSC1	-0.41	0.0023
K_L	SSC2	-0.37	0.0064
K_L	TFW	-0.49	0.0002
Na_L	NFp	-0.43	0.0009
Na_L	TFW	-0.52	0.0001
NFp	SSC1	0.59	<0.0001
NFp	SSC2	0.42	0.0019
NFp	TFW	0.92	<0.0001
SSC1	SSC2	0.77	<0.0001
SSC1	TFW	0.44	0.0009
TChl	TFW	0.32	0.0170

Table S3. Trait abbreviations and heritability estimates (H2_C and H2_S for control and salinity treatments, respectively) and *p* values for the significant effects in the mixed model analysis of evaluated traits in the NG experiment. G and E denote genotype and salinity effects, respectively. ns: non-significant.

Trait abbreviation	Trait	H2_C	H2_S	G	E	GxE
C_L	Total C leaf concentration (g/100g)	0.3501	0.4728	<0.0001	ns	0.0041
C_R	Total C root concentration (g/100g)	0.3161	0.2568	0.0001	ns	0.0002
Ca_L	Leaf Ca concentration (ppm)	0.3691	0.4866	<0.0001	ns	ns
Ca_R	Root Ca concentration (ppm)	0.4375	0.1559	<0.0001	<0.0001	0.004
Cl_L	Leaf Cl concentration (mg L^{-1})	0.7354	0.644	<0.0001	<0.0001	<0.0001
Cl_R	Root Cl concentration (mg L^{-1})	0.3183	0.3667	<0.0001	<0.0001	<0.0001
ClR-ClL/ClR	$[Cl]_{root}$ –$[Cl]_{leaf}$)/$[Cl]_{root}$	0.6325	0.5306	<0.0001	<0.0001	<0.0001
Fe_L	Leaf Fe concentration (ppm)	0	0.0397	0.0103	ns	ns
Fe_R	Root Fe concentration (ppm)	0.3815	0.0689	<0.0001	<0.0001	0.0024
FRDW	Fine root dry weight (g)	0.1948	0.2155	<0.0001	0.0002	0.0187
K_L	Leaf K concentration (ppm)	0.4401	0.4073	<0.0001	<0.0001	0.0001
K_R	Root K concentration (ppm)	0.3158	0.3355	<0.0001	<0.0001	0.0015
N_L	Total N leaf concentration (g/100g)	0.1961	0.5307	<0.0001	<0.0001	0.0357
N_R	Total N root concentration (g/100g)	0.4409	0.1658	<0.0001	<0.0001	0.0001
Na_L	Leaf Na concentration (ppm)	0.4736	0.4543	<0.0001	<0.0001	<0.0001
Na_R	Root Na concentration (ppm)	0.6232	0.3492	<0.0001	<0.0001	0.0256
TRDW	Total root dry weight (g)	0.1706	0.2448	<0.0001	ns	ns

Table S4. Spearman coefficients (r) of significantly correlated traits (p<0.05) between traits evaluated in the NG experiment under control and salinity conditions, and for each trait under both salinity conditions.

Trait 1_C	Trait 2_C	r	p-value
(CaR-CaL/CaR)	Ca_L	-0.57	0.0001
(CaR-CaL/CaR)	Ca_R	0.67	<0.0001
(CaR-CaL/CaR)	d(CaR-CaL/CaR)	0.53	0.0003
(CaR-CaL/CaR)	dCa_L	0.34	0.0262
(CaR-CaL/CaR)	Na_R	0.44	0.0038
(ClR-ClL/ClR)	Ca_L	-0.38	0.0122
(ClR-ClL/ClR)	Cl_R	0.58	0.0001
(ClR-ClL/ClR)	K_R	0.40	0.0085
C_L	(CaR-CaL/CaR)	0.36	0.0182
C_L	K_L	-0.58	0.0001
C_R	Fe_R	-0.62	<0.0001
Ca_L	Fe_L	0.39	0.0101
Ca_L	K_R	-0.33	0.0371
Ca_R	Fe_R	0.32	0.0416
Ca_R	Na_R	0.46	0.0032
Cl_L	(ClR-ClL/ClR)	-0.75	<0.0001
Cl_L	Ca_L	0.41	0.0076
Cl_R	dCl_R	-0.45	0.0027
Cl_R	K_R	0.33	0.0345
Cl_R	Na_R	0.47	0.0016
d(CaR-CaL/CaR)	Ca_R	0.69	<0.0001
d(CaR-CaL/CaR)	Fe_R	0.35	0.0213
d(CaR-CaL/CaR)	Na_R	0.52	0.0004
d(ClR-ClL/ClR)	Ca_R	-0.46	0.0019
d(ClR-ClL/ClR)	d(CaR-CaL/CaR)	-0.56	0.0001

Trait 1_S	Trait 2_S	r	p-value
(CaR-CaL/CaR)	Ca_L	-0.87	<0.0001
(CaR-CaL/CaR)	Ca_R	0.56	0.0001
(CaR-CaL/CaR)	d(CaR-CaL/CaR)	-0.42	0.006
(CaR-CaL/CaR)	dCa_L	-0.47	0.0018
(CaR-CaL/CaR)	dCa_R	0.45	0.0029
(CaR-CaL/CaR)	Fe_R	0.33	0.0347
(CaR-CaL/CaR)	K_R	0.34	0.0278
(ClR-ClL/ClR)	(CaR-CaL/CaR)	0.48	0.0013
(ClR-ClL/ClR)	C_R	-0.31	0.0455
(ClR-ClL/ClR)	Ca_L	-0.50	0.0009
(ClR-ClL/ClR)	Cl_R	0.46	0.0021
(ClR-ClL/ClR)	d(CaR-CaL/CaR)	-0.56	0.0001
(ClR-ClL/ClR)	d(ClR-ClL/ClR)	1.00	<0.0001
(ClR-ClL/ClR)	dCa_L	-0.39	0.0097
(ClR-ClL/ClR)	dCa_R	0.61	<0.0001
(ClR-ClL/ClR)	dCl_L	-0.85	<0.0001
(ClR-ClL/ClR)	dCl_R	0.48	0.0013
(ClR-ClL/ClR)	K_R	0.57	0.0001
(ClR-ClL/ClR)	N_L	0.67	<0.0001
(ClR-ClL/ClR)	Na_L	-0.56	0.0001
C_L	(CaR-CaL/CaR)	0.35	0.0243
C_L	Ca_L	-0.37	0.0152
C_L	K_L	-0.66	<0.0001
C_R	dCa_L	-0.39	0.0114
C_R	Fe_R	-0.42	0.0052

Trait_C	Trait_S	r	p-value
(CaR-CaL/CaR)	(CaR-CaL/CaR)	0.45	0.0028
C_L	C_L	0.46	0.0019
Ca_L	Ca_L	0.64	<0.0001
Ca_R	Ca_R	0.42	0.0076
Cl_R	Cl_R	0.40	0.0081
FRDW	FRDW	0.36	0.0191
K_L	K_L	0.32	0.0395
N_L	N_L	0.32	0.0385
Na_L	Na_L	0.60	<0.0001
Na_R	Na_R	0.66	<0.0001
TRDW	TRDW	0.57	0.0003

d(ClR-ClL/ClR)	dCa_L	-0.41	0.0072	C_R	K_R	-0.49	0.0009
d(ClR-ClL/ClR)	dCa_R	0.60	<0.0001	Ca_L	Fe_L	0.33	0.0338
d(ClR-ClL/ClR)	dCl_R	0.48	0.0012	Ca_L	Fe_R	-0.33	0.0374
d(ClR-ClL/ClR)	Fe_R	-0.34	0.0294	Ca_L	K_L	0.32	0.0382
d(ClR-ClL/ClR)	N_R	-0.38	0.0141	Ca_R	K_R	0.43	0.0064
dCa_L	Ca_R	0.36	0.0184	Cl_L	(CaR-CaL/CaR)	-0.49	0.001
dCa_L	d(CaR-CaL/CaR)	0.70	<0.0001	Cl_L	(ClR-ClL/ClR)	-0.96	<0.0001
dCa_L	K_R	0.36	0.0177	Cl_L	Ca_L	0.54	0.0006
dCa_L	Na_R	0.53	0.0003	Cl_L	d(CaR-CaL/CaR)	0.59	<0.0001
dCa_R	Ca_L	-0.37	0.0149	Cl_L	d(ClR-ClL/ClR)	-0.96	<0.0001
dCa_R	Ca_R	-0.71	<0.0001	Cl_L	dCa_L	0.45	0.003
dCa_R	d(CaR-CaL/CaR)	-0.80	<0.0001	Cl_L	dCa_R	-0.58	0.0001
dCa_R	Fe_L	-0.33	0.0356	Cl_L	dCl_L	0.87	<0.0001
dCa_R	Fe_R	-0.32	0.0373	Cl_L	dCl_R	-0.41	0.0069
dCa_R	Na_L	-0.35	0.0219	Cl_L	Fe_L	0.35	0.0267
dCa_R	Na_R	-0.33	0.0307	Cl_L	K_L	0.34	0.0301
dCl_L	Ca_R	0.33	0.0358	Cl_L	K_R	-0.53	0.0007
dCl_L	d(CaR-CaL/CaR)	0.50	0.0007	Cl_L	N_L	-0.61	<0.0001
dCl_L	d(ClR-ClL/ClR)	-0.83	<0.0001	Cl_L	Na_L	0.57	0.0003
dCl_L	dCa_L	0.39	0.0108	Cl_R	C_R	-0.47	0.0018
dCl_L	dCa_R	-0.46	0.0024	Cl_R	d(ClR-ClL/ClR)	0.46	0.002
dCl_L	dCl_R	-0.47	0.0018	Cl_R	dCa_R	0.31	0.0464
dCl_R	Ca_R	-0.31	0.0466	Cl_R	dCl_L	-0.36	0.0192
dCl_R	d(CaR-CaL/CaR)	-0.33	0.0335	Cl_R	dCl_R	0.60	<0.0001
dCl_R	dCa_R	0.43	0.0042	Cl_R	K_R	0.56	0.0001
dCl_R	Na_L	-0.35	0.0232	Cl_R	N_L	0.52	0.0005
FRDW	(CaR-CaL/CaR)	-0.33	0.0357	Cl_R	Na_R	0.58	0.0001
FRDW	Ca_L	0.46	0.0024	d(CaR-CaL/CaR)	Ca_L	0.49	0.0009

FRDW	N_L	-0.35	0.0244
K_L	K_R	-0.39	0.012
N_L	C_L	-0.53	0.0004
N_L	Ca_R	-0.35	0.0224
N_L	dCa_L	-0.34	0.0269
N_L	K_R	0.33	0.0327
N_L	N_R	-0.32	0.0399
N_L	Na_R	-0.38	0.0123
N_R	(CaR-CaL/CaR)	0.47	0.0018
N_R	Ca_R	0.62	<0.0001
N_R	d(CaR-CaL/CaR)	0.35	0.0212
N_R	dCa_R	-0.44	0.0039
N_R	Na_R	0.40	0.0084
Na_L	Ca_R	0.31	0.0455
Na_L	K_R	-0.34	0.0263
TRDW	Ca_L	0.52	0.0008
TRDW	d(ClR-ClL/ClR)	-0.34	0.0294
TRDW	FRDW	0.82	<0.0001
TRDW	K_R	-0.45	0.0042
TRDW	N_L	-0.50	0.0007

d(CaR-CaL/CaR)	Fe_L	0.36	0.0198
d(CaR-CaL/CaR)	K_R	-0.40	0.0087
d(ClR-ClL/ClR)	(CaR-CaL/CaR)	0.46	0.002
d(ClR-ClL/ClR)	Ca_L	-0.49	0.001
d(ClR-ClL/ClR)	d(CaR-CaL/CaR)	-0.56	0.0001
d(ClR-ClL/ClR)	dCa_L	-0.41	0.0072
d(ClR-ClL/ClR)	dCa_R	0.60	<0.0001
d(ClR-ClL/ClR)	dCl_R	0.48	0.0012
d(ClR-ClL/ClR)	K_R	0.56	0.0001
d(ClR-ClL/ClR)	N_L	0.67	<0.0001
d(ClR-ClL/ClR)	Na_L	-0.56	0.0001
dCa_L	Ca_L	0.64	<0.0001
dCa_L	d(CaR-CaL/CaR)	0.70	<0.0001
dCa_L	K_L	0.36	0.0203
dCa_L	Na_R	0.34	0.0282
dCa_R	Ca_L	-0.40	0.0081
dCa_R	d(CaR-CaL/CaR)	-0.80	<0.0001
dCa_R	Fe_L	-0.35	0.0213
dCa_R	K_R	0.57	0.0001
dCa_R	Na_L	-0.45	0.0031
dCl_L	(CaR-CaL/CaR)	-0.37	0.0168
dCl_L	Ca_L	0.39	0.0124
dCl_L	d(CaR-CaL/CaR)	0.50	0.0007
dCl_L	d(ClR-ClL/ClR)	-0.83	<0.0001
dCl_L	dCa_L	0.39	0.0108
dCl_L	dCa_R	-0.46	0.0024
dCl_L	dCl_R	-0.47	0.0018
dCl_L	K_R	-0.45	0.0038

dCl_L	N_L	-0.46	0.002
dCl_L	Na_L	0.50	0.0015
dCl_R	d(CaR-CaL/CaR)	-0.33	0.0335
dCl_R	dCa_R	0.43	0.0042
dCl_R	K_R	0.47	0.0015
dCl_R	N_L	0.45	0.0027
dCl_R	Na_L	-0.42	0.006
FRDW	(CaR-CaL/CaR)	-0.48	0.0011
FRDW	(ClR-ClL/ClR)	-0.48	0.0012
FRDW	Ca_L	0.36	0.0179
FRDW	Ca_R	-0.34	0.0266
FRDW	Cl_L	0.43	0.0041
FRDW	Cl_R	-0.42	0.0058
FRDW	d(ClR-ClL/ClR)	-0.48	0.0015
FRDW	dCa_R	-0.50	0.0008
FRDW	dCl_R	-0.35	0.0246
FRDW	K_R	-0.50	0.0008
FRDW	N_L	-0.58	0.0001
N_L	(CaR-CaL/CaR)	0.34	0.0291
N_L	d(CaR-CaL/CaR)	-0.47	0.0015
N_L	dCa_R	0.53	0.0003
N_L	K_R	0.65	<0.0001
N_L	Na_L	-0.39	0.01
N_R	K_R	0.33	0.0303
Na_L	K_R	-0.49	0.0017
TRDW	(CaR-CaL/CaR)	-0.37	0.0172
TRDW	(ClR-ClL/ClR)	-0.52	0.0005
TRDW	Cl_L	0.44	0.0051

TRDW	Cl_R	-0.53	0.0003
TRDW	d(ClR-ClL/ClR)	-0.51	0.0005
TRDW	dCa_R	-0.48	0.0013
TRDW	dCl_R	-0.46	0.0021
TRDW	FRDW	0.95	<0.0001
TRDW	K_R	-0.55	0.0005
TRDW	N_L	-0.58	0.0001
TRDW	Na_L	0.32	0.0423

Table S5. List of candidate genes (mRNA) that might be involved in leaf Cl$^-$ exclusion/accumulation at *LCl-6* downloaded from the *P. trifoliata* genome database at https://phytozome.jgi.doe.gov using markers 15R,750 and CR28,270. The starting physical position (in bp), description, and comparison(s) where differentially expressed genes (DEGs) have been detected, are provided. Comparisons 107_15 vs 90_15, and (107_15 + 107_0) vs (90_15 + 90_0) are coded as 2 and 3, respectively.

mRNA	start	DEG in	Description
Ptrif.0006s0368.1	7872523		K02575 - MFS transporter, NNP family, nitrate/nitrite transporter (NRT, narK, nrtP, nasA)
Ptrif.0006s0402.1	8525565		PTHR10217//PTHR10217:SF494 - VOLTAGE AND LIGAND GATED POTASSIUM CHANNEL
Ptrif.0006s0417.1	8818622		PTHR19139//PTHR19139:SF167 - AQUAPORIN TRANSPORTER // AQUAPORIN PIP2-1-RELATED
Ptrif.0006s0418.2	8821986	3	PTHR19139:SF167 - AQUAPORIN PIP2-1-RELATED
Ptrif.0006s0419.1	8843501		PTHR19139//PTHR19139:SF167 - AQUAPORIN TRANSPORTER // AQUAPORIN PIP2-1-RELATED
Ptrif.0006s0420.1	8850298		K02152 - V-type H+-transporting ATPase subunit G (ATPeV1G, ATP6G)
Ptrif.0006s0539.1	10505139	3	3.6.3.29 - Molybdate-transporting ATPase
Ptrif.0006s0540.2	10512840	3	PTHR19241//PTHR19241:SF258 - ATP-BINDING CASSETTE TRANSPORTER // ABC TRANSPORTER G FAMILY MEMBER 17-RELATED
Ptrif.0006s0541.1	10535146	3	PTHR19241//PTHR19241:SF258 - ATP-BINDING CASSETTE TRANSPORTER // ABC TRANSPORTER G FAMILY MEMBER 17-RELATED
Ptrif.0006s0601.1	11185093	3	PTHR32468:SF10 - CATION/H(+) ANTIPORTER 20
Ptrif.0006s0604.2	11267097		PTHR32468:SF10 - CATION/H(+) ANTIPORTER 20
Ptrif.0006s0606.2	11316426		PTHR32468:SF10 - CATION/H(+) ANTIPORTER 20
Ptrif.0006s0610.1	11359669	2, 3	PTHR19444 - UNC-93 RELATED - Major facilitator superfamily protein
Ptrif.0006s0696.2	12305174	2, 3	K07300 - Ca2+:H+ antiporter (chaA, CAX)
Ptrif.0006s0734.1	12658870		PTHR11654//PTHR11654:SF181 - OLIGOPEPTIDE TRANSPORTER-RELATED // PROTEIN NRT1/ PTR FAMILY 2.8 (NPF2.3)
Ptrif.0006s2462.1	12728591	3	PTHR11654:SF79 - PROTEIN NRT1/ PTR FAMILY 5.5-RELATED (NPF5.12)
Ptrif.0006s0803.2	13122768		PTHR11662//PTHR11662:SF235 - SODIUM-DEPENDENT PHOSPHATE TRANSPORTERS // ANION TRANSPORTER 3
Ptrif.0006s0814.5	13277480	3	PTHR11654//PTHR11654:SF125 - OLIGOPEPTIDE TRANSPORTER-RELATED (NPF8.1)
Ptrif.0006s0815.1	13284465	3	PTHR11654//PTHR11654:SF125 - OLIGOPEPTIDE TRANSPORTER-RELATED (NPF8.2)
Ptrif.0006s0823.1	13354423	2, 3	PTHR11654:SF79 - PROTEIN NRT1/ PTR FAMILY 5.5-RELATED (NPF5.9)
Ptrif.0006s0824.1	13368702		K14638 - solute carrier family 15 (peptide/histidine transporter) (NPF5.8)
Ptrif.0006s2370.1	13372277		PTHR11654:SF79 - PROTEIN NRT1/ PTR FAMILY 5.5-RELATED (NPF5.10)
Ptrif.0006s0828.1	13400568		PTHR19139//PTHR19139:SF169 - AQUAPORIN TRANSPORTER // AQUAPORIN PIP1-4-RELATED

Table S6. Summary of raw, clean and mapped reads by library, including the full-sib and NaCl concentrations used.

Library	Full-sib	NaCl (mM)	Raw reads	Clean reads		Mapping (%)	
				Amount	%	*C. clementina*	*P. trifoliata*
NGS083-20-1_S1	107	0	12943104	10946681	84,58	84,60	69,12
NGS083-20-2_S2	107	0	11786382	9975424	84,64	85,30	71,41
NGS083-20-3_S3	107	0	12716482	10781816	84,79	84,23	69,00
NGS083-20-4_S4	90	0	10831538	9102085	84,03	79,50	61,25
NGS083-20-5_S5	90	0	11304859	9596465	84,89	84,40	68,19
NGS083-20-6_S6	90	0	12355784	10484140	84,85	83,18	67,30
NGS083-20-7_S7	107	15	11227180	9486618	84,50	83,94	68,49
NGS083-20-8_S8	107	15	12247759	10352414	84,52	84,82	70,48
NGS083-20-9_S9	107	15	11723647	9889443	84,35	83,97	69,38
NGS083-20-10_S10	90	15	12244396	10294030	84,07	84,66	69,92
NGS083-20-11_S11	90	15	11904751	9826470	82,54	84,63	69,87
NGS083-20-12_S12	90	15	12886702	10831352	84,05	83,50	69,16

Table S7. List of DEGs in *LCl-6* coded by expressed sequence (ESR) and transcript (mRNA), using the *C. clementina* genome as reference, per comparison. Information on gene physical position (start), fold change expression (logFC), adjusted *p* value, and annotation, when known, is provided.

Comparison	ESRs	mRNA	logFC	padj	Start	Candi-date	Anotation
(107-15 + 107-0) vs (90-15 + 90-0)	ESR42000	Ciclev10013303m	-1.566	0.066	10032778		
(107-15 + 107-0) vs (90-15 + 90-0)	ESR42011	Ciclev10012331m	-1.442	0.023	10082734		chloroplast RNA-binding protein 29
(107-15 + 107-0) vs (90-15 + 90-0)	ESR42010	Ciclev10012331m	-1.301	0.020	10082734		chloroplast RNA-binding protein 29
(107-15 + 107-0) vs (90-15 + 90-0)	ESR42008	Ciclev10012331m	-1.272	0.052	10082734		chloroplast RNA-binding protein 29
(107-15 + 107-0) vs (90-15 + 90-0)	ESR42009	Ciclev10012331m	-1.393	0.032	10082734		chloroplast RNA-binding protein 29
(107-15 + 107-0) vs (90-15 + 90-0)	ESR42007	Ciclev10012331m	-1.177	0.030	10082734		chloroplast RNA-binding protein 29
(107-15 + 107-0) vs (90-15 + 90-0)	ESR42028	Ciclev10012375m	2.153	0.020	10419232	PIP2-1	plasma membrane intrinsic protein 2
(107-15 + 107-0) vs (90-15 + 90-0)	ESR42033	Ciclev10011116m	2.210	0.063	10438273		CRINKLY4 related 1
(107-15 + 107-0) vs (90-15 + 90-0)	ESR42034	Ciclev10013160m	-2.043	0.005	10448885		
107-0 vs 90-0	ESR42034	Ciclev10013160m	-1.393	0.039	10448885		
(107-15 + 107-0) vs (90-15 + 90-0)	ESR42035	Ciclev10013919m	-1.721	0.052	10451057		Protein kinase superfamily protein
(107-15 + 107-0) vs (90-15 + 90-0)	ESR42044	Ciclev10012588m	3.432	0.052	10597545		FASCICLIN-like arabinogalactan-protein 11
(107-15 + 107-0) vs (90-15 + 90-0)	ESR42066	Ciclev10012170m	1.199	0.093	11043782		Peroxidase superfamily protein
(107-15 + 107-0) vs (90-15 + 90-0)	ESR42073	Ciclev10012558m	1.102	0.081	11112519		
(107-15 + 107-0) vs (90-15 + 90-0)	ESR42081	Ciclev10011171m	4.390	0.068	11177534		Leucine-rich repeat receptor-like protein kinase
(107-15 + 107-0) vs (90-15 + 90-0)	ESR42096	Ciclev10012036m	3.305	0.091	11374942		Nucleotide-diphospho-sugar transferases
(107-15 + 107-0) vs (90-15 + 90-0)	ESR42097	Ciclev10011643m	2.612	0.045	11401278		IQ-domain 2
(107-15 + 107-0) vs (90-15 + 90-0)	ESR42098	Ciclev10011643m	2.741	0.037	11401278		IQ-domain 2
(107-15 + 107-0) vs (90-15 + 90-0)	ESR42117	Ciclev10013573m	2.951	0.088	11554084		minichromosome maintenance 8
107-0 vs 90-0	ESR42117	Ciclev10013573m	2.803	0.089	11554084		minichromosome maintenance 8
(107-15 + 107-0) vs (90-15 + 90-0)	ESR42136	Ciclev10013509m	-4.695	0.052	11734007		target of early activation tagged (EAT) 2
(107-15 + 107-0) vs (90-15 + 90-0)	ESR42159	Ciclev10011660m	0.994	0.037	11954097		WRKY family transcription factor family protein
(107-15 + 107-0) vs (90-15 + 90-0)	ESR42165	Ciclev10012291m	-1.337	0.016	12023785		ubiquitin 4
(107-15 + 107-0) vs (90-15 + 90-0)	ESR42179	Ciclev10011952m	-1.131	0.079	12224206		integral membrane TerC family protein

(107-15 + 107-0) vs (90-15 + 90-0)	ESR42191	Ciclev10012410m	3.830	0.054	12270372		RING/U-box superfamily protein
(107-15 + 107-0) vs (90-15 + 90-0)	ESR42210	Ciclev10013542m	3.253	0.094	12407037		O-methyltransferase 1
(107-15 + 107-0) vs (90-15 + 90-0)	ESR42211	Ciclev10011291m	4.015	0.003	12440026		Fatty acid hydroxylase superfamily
107-0 vs 90-0	ESR42211	Ciclev10011291m	1.746	0.089	12440026		Fatty acid hydroxylase superfamily
107-15 vs 90-15	ESR42211	Ciclev10011291m	2.268	0.038	12440026		Fatty acid hydroxylase superfamily
(107-15 + 107-0) vs (90-15 + 90-0)	ESR42213	Ciclev10013485m	3.770	0.075	12469611	ABCG-17	ABC-2 type transporter family protein
(107-15 + 107-0) vs (90-15 + 90-0)	ESR42214	Ciclev10011167m	1.795	0.030	12478048	ABCG-17	ABC-2 type transporter family protein
(107-15 + 107-0) vs (90-15 + 90-0)	ESR42215	Ciclev10011147m	2.226	0.054	12497442	ABCG-17	ABC-2 type transporter family protein
(107-15 + 107-0) vs (90-15 + 90-0)	ESR42216	Ciclev10010954m	0.808	0.075	12508551		endomembrane-type CA-ATPase 4
(107-15 + 107-0) vs (90-15 + 90-0)	ESR42218	Ciclev10011715m	-2.919	0.001	12551908		HXXXD-type acyl-transferase family protein
107-0 vs 90-0	ESR42218	Ciclev10011715m	-1.419	0.050	12551908		HXXXD-type acyl-transferase family protein
107-15 vs 90-15	ESR42218	Ciclev10011715m	-1.500	0.029	12551908		HXXXD-type acyl-transferase family protein
(107-15 + 107-0) vs (90-15 + 90-0)	ESR42219	Ciclev10012982m	5.110	0.054	12558711		
(107-15 + 107-0) vs (90-15 + 90-0)	ESR42230	Ciclev10013809m	-3.548	0.094	12694282		Ankyrin repeat family protein
(107-15 + 107-0) vs (90-15 + 90-0)	ESR42232	Ciclev10011951m	-1.376	0.037	12700076		Pectin lyase-like superfamily protein
(107-15 + 107-0) vs (90-15 + 90-0)	ESR42242	Ciclev10012060m	-1.030	0.018	12883561		Tetratricopeptide repeat (TPR)-like protein
(107-15 + 107-0) vs (90-15 + 90-0)	ESR42249	Ciclev10010956m	2.866	0.088	12916688		ATP binding microtubule motor family protein
(107-15 + 107-0) vs (90-15 + 90-0)	ESR42257	Ciclev10012723m	-1.234	0.052	13032633		pyrophosphorylase 4
(107-15 + 107-0) vs (90-15 + 90-0)	ESR42258	Ciclev10012723m	-1.308	0.048	13032633		pyrophosphorylase 4
(107-15 + 107-0) vs (90-15 + 90-0)	ESR42262	Ciclev10013673m	-0.914	0.088	13071301		
(107-15 + 107-0) vs (90-15 + 90-0)	ESR42270	Ciclev10012475m	-0.839	0.095	13208438		
(107-15 + 107-0) vs (90-15 + 90-0)	ESR42280	Ciclev10011060m	1.426	0.097	13305253		cation/H+ exchanger 20
(107-15 + 107-0) vs (90-15 + 90-0)	ESR42288	Ciclev10013572m	4.029	0.063	13444058		
(107-15 + 107-0) vs (90-15 + 90-0)	ESR42290	Ciclev10011745m	2.067	0.001	13446344	MFS	Major facilitator superfamily protein
(107-15 + 107-0) vs (90-15 + 90-0)	ESR42291	Ciclev10011745m	1.679	0.001	13446344	MFS	Major facilitator superfamily protein

(107-15 + 107-0) vs (90-15 + 90-0)	ESR42289	Ciclev10011745m	1.864	0.003	13446344	MFS	Major facilitator superfamily protein
107-0 vs 90-0	ESR42290	Ciclev10011745m	0.872	0.089	13446344	MFS	Major facilitator superfamily protein
107-0 vs 90-0	ESR42291	Ciclev10011745m	0.793	0.067	13446344	MFS	Major facilitator superfamily protein
107-15 vs 90-15	ESR42290	Ciclev10011745m	1.195	0.029	13446344	MFS	Major facilitator superfamily protein
107-15 vs 90-15	ESR42291	Ciclev10011745m	0.886	0.038	13446344	MFS	Major facilitator superfamily protein
107-15 vs 90-15	ESR42289	Ciclev10011745m	1.214	0.029	13446344	MFS	Major facilitator superfamily protein
(107-15 + 107-0) vs (90-15 + 90-0)	ESR42293	Ciclev10013882m	3.598	0.088	13489604		NAD(P)-binding Rossmann-fold superfamily protein
(107-15 + 107-0) vs (90-15 + 90-0)	ESR42295	Ciclev10012849m	-0.805	0.075	13498177		Ribosomal protein L12
(107-15 + 107-0) vs (90-15 + 90-0)	ESR42312	Ciclev10013628m	2.304	0.067	13645123		Transducin/WD40 repeat-like superfamily protein
(107-15 + 107-0) vs (90-15 + 90-0)	ESR42320	Ciclev10013810m	-1.591	0.068	13745347		Protein of unknown function (DUF3741)
(107-15 + 107-0) vs (90-15 + 90-0)	ESR42330	Ciclev10012089m	-1.685	0.001	14087181		myb-like HTH transcriptional regulator
107-0 vs 90-0	ESR42330	Ciclev10012089m	-1.056	0.025	14087181		myb-like HTH transcriptional regulator
(107-15 + 107-0) vs (90-15 + 90-0)	ESR42348	Ciclev10012381m	-1.476	0.052	14277934		
(107-15 + 107-0) vs (90-15 + 90-0)	ESR42346	Ciclev10012381m	-1.791	0.037	14277934		
(107-15 + 107-0) vs (90-15 + 90-0)	ESR42368	Ciclev10013090m	1.293	0.037	14397047		
(107-15 + 107-0) vs (90-15 + 90-0)	ESR42374	Ciclev10011334m	1.446	0.096	14451669		Pathogenesis-related thaumatin protein
(107-15 + 107-0) vs (90-15 + 90-0)	ESR42375	Ciclev10011734m	-0.839	0.052	14472657		Tetratricopeptide repeat (TPR)-like y protein
(107-15 + 107-0) vs (90-15 + 90-0)	ESR42382	Ciclev10013799m	1.320	0.052	14498799		Concanavalin A-like lectin protein kinase family protein
(107-15 + 107-0) vs (90-15 + 90-0)	ESR42385	Ciclev10011508m	3.325	0.052	14524326		Protein of unknown function (DUF604)
(107-15 + 107-0) vs (90-15 + 90-0)	ESR42394	Ciclev10012494m	-3.160	0.007	14604868		NAD(P)-binding Rossmann-fold superfamily protein
107-0 vs 90-0	ESR42394	Ciclev10012494m	-2.017	0.050	14604868		NAD(P)-binding Rossmann-fold superfamily protein
(107-15 + 107-0) vs (90-15 + 90-0)	ESR42395	Ciclev10012221m	-2.130	0.088	14625884		NAD(P)-binding Rossmann-fold superfamily protein
(107-15 + 107-0) vs (90-15 + 90-0)	ESR42406	Ciclev10011164m	1.694	0.037	14694513		basic helix-loop-helix (bHLH) DNA-binding prot.
(107-15 + 107-0) vs (90-15 + 90-0)	ESR42407	Ciclev10013212m	3.506	0.004	14721970		
107-0 vs 90-0	ESR42407	Ciclev10013212m	1.974	0.050	14721970		
(107-15 + 107-0) vs (90-15 + 90-0)	ESR42413	Ciclev10012040m	-1.437	0.007	14754810		Glucose-1-phosphate adenylyltransferase prot.
(107-15 + 107-0) vs (90-15 + 90-0)	ESR42412	Ciclev10012040m	-1.057	0.063	14754810		Glucose-1-phosphate adenylyltransferase prot.
107-0 vs 90-0	ESR42412	Ciclev10012040m	-0.895	0.089	14754810		Glucose-1-phosphate adenylyltransferase prot.

(107-15 + 107-0) vs (90-15 + 90-0)	ESR42418	Ciclev10012242m	2.582	0.005	14770122		NAD(P)-linked oxidoreductase superfamily protein
(107-15 + 107-0) vs (90-15 + 90-0)	ESR42417	Ciclev10012242m	1.734	0.068	14770122		NAD(P)-linked oxidoreductase superfamily protein
107-15 vs 90-15	ESR42418	Ciclev10012242m	1.804	0.029	14770122		NAD(P)-linked oxidoreductase superfamily protein
(107-15 + 107-0) vs (90-15 + 90-0)	ESR42443	Ciclev10010967m	-3.057	0.020	14988237		Leucine-rich repeat protein kinase family protein
107-0 vs 90-0	ESR42443	Ciclev10010967m	-1.953	0.089	14988237		Leucine-rich repeat protein kinase family protein
(107-15 + 107-0) vs (90-15 + 90-0)	ESR42454	Ciclev10011268m	0.887	0.067	15070590		POX (plant homeobox) family protein
(107-15 + 107-0) vs (90-15 + 90-0)	ESR42456	Ciclev10011341m	-1.311	0.011	15116860	NPF5.12	Major facilitator superfamily protein
107-0 vs 90-0	ESR42456	Ciclev10011341m	-0.810	0.089	15116860	NPF5.12	Major facilitator superfamily protein
(107-15 + 107-0) vs (90-15 + 90-0)	ESR42462	Ciclev10013234m	-1.847	0.048	15151156		
107-0 vs 90-0	ESR42462	Ciclev10013234m	-1.432	0.089	15151156		
(107-15 + 107-0) vs (90-15 + 90-0)	ESR42470	Ciclev10013132m	3.992	0.001	15164058		Bifunctional inhibitor/lipid-transfer protein
107-0 vs 90-0	ESR42470	Ciclev10013132m	1.706	0.060	15164058		Bifunctional inhibitor/lipid-transfer protein
107-15 vs 90-15	ESR42470	Ciclev10013132m	2.286	0.029	15164058		Bifunctional inhibitor/lipid-transfer protein
(107-15 + 107-0) vs (90-15 + 90-0)	ESR42487	Ciclev10012250m	-1.145	0.039	15275665		Arabidopsis Inositol phosphorylceramide Synthase1
(107-15 + 107-0) vs (90-15 + 90-0)	ESR42494	Ciclev10011384m	1.500	0.063	15311565		laccase 7
(107-15 + 107-0) vs (90-15 + 90-0)	ESR42498	Ciclev10011375m	3.116	0.016	15354935		laccase 7
107-0 vs 90-0	ESR42498	Ciclev10011375m	1.898	0.089	15354935		laccase 7
(107-15 + 107-0) vs (90-15 + 90-0)	ESR42500	Ciclev10012045m	2.052	0.053	15377566		Concanavalin A-like lectin family protein
(107-15 + 107-0) vs (90-15 + 90-0)	ESR42505	Ciclev10011279m	2.927	0.016	15404434		O-fucosyltransferase family protein
107-0 vs 90-0	ESR42505	Ciclev10011279m	1.803	0.091	15404434		O-fucosyltransferase family protein
(107-15 + 107-0) vs (90-15 + 90-0)	ESR42515	Ciclev10012753m	1.259	0.088	15443543		Reticulon family protein
(107-15 + 107-0) vs (90-15 + 90-0)	ESR42530	Ciclev10013166m	-1.233	0.039	15537914		Protein of unknown function (DUF962)
(107-15 + 107-0) vs (90-15 + 90-0)	ESR42534	Ciclev10013252m	5.059	0.013	15548237		
107-0 vs 90-0	ESR42534	Ciclev10013252m	3.476	0.067	15548237		
(107-15 + 107-0) vs (90-15 + 90-0)	ESR42535	Ciclev10012894m	3.209	0.052	15553485		
(107-15 + 107-0) vs (90-15 + 90-0)	ESR42542	Ciclev10011239m	1.831	0.068	15703559		Protein of unknown function (DUF740)
(107-15 + 107-0) vs (90-15 + 90-0)	ESR42547	Ciclev10011381m	2.177	0.005	15747188	NPF8.1	peptide transporter 1

(107-15 + 107-0) vs (90-15 + 90-0)	ESR42546	Ciclev10011381m	2.424	0.001	15747188	NPF8.1	peptide transporter 1
107-0 vs 90-0	ESR42547	Ciclev10011381m	1.460	0.050	15747188	NPF8.1	peptide transporter 1
107-0 vs 90-0	ESR42546	Ciclev10011381m	1.407	0.025	15747188	NPF8.1	peptide transporter 1
(107-15 + 107-0) vs (90-15 + 90-0)	ESR42548	Ciclev10013821m	6.813	0.088	15754314	NPF8.2	peptide transporter 1
(107-15 + 107-0) vs (90-15 + 90-0)	ESR42549	Ciclev10011400m	3.506	0.076	15761841		Laccase/Diphenol oxidase family protein
(107-15 + 107-0) vs (90-15 + 90-0)	ESR42550	Ciclev10012285m	1.893	0.005	15774976		Duplicated homeodomain-like superfamily protein
107-0 vs 90-0	ESR42550	Ciclev10012285m	1.129	0.060	15774976		Duplicated homeodomain-like superfamily protein
(107-15 + 107-0) vs (90-15 + 90-0)	ESR42558	Ciclev10013337m	7.755	0.003	15843855	NPF5.9	proton-dependent oligopeptide transport (POT) prot.
107-0 vs 90-0	ESR42558	Ciclev10013337m	3.478	0.060	15843855	NPF5.9	proton-dependent oligopeptide transport (POT) prot.
107-15 vs 90-15	ESR42558	Ciclev10013337m	4.277	0.038	15843855	NPF5.9	proton-dependent oligopeptide transport (POT) prot.
(107-15 + 107-0) vs (90-15 + 90-0)	ESR42562	Ciclev10011536m	2.170	0.054	15879061		glycerol-3-phosphate acyltransferase 6
(107-15 + 107-0) vs (90-15 + 90-0)	ESR42573	Ciclev10013652m	-1.546	0.016	16023582		Leucine-rich repeat protein kinase family protein
(107-15 + 107-0) vs (90-15 + 90-0)	ESR42577	Ciclev10013537m	-3.982	0.063	16084538		Leucine-rich repeat protein kinase family protein
(107-15 + 107-0) vs (90-15 + 90-0)	ESR42581	Ciclev10011409m	-0.975	0.057	16154922		Carbohydrate-binding-like fold
(107-15 + 107-0) vs (90-15 + 90-0)	ESR42583	Ciclev10011409m	-1.160	0.052	16154922		Carbohydrate-binding-like fold
(107-15 + 107-0) vs (90-15 + 90-0)	ESR42596	Ciclev10012370m	2.692	0.053	16218093		light harvesting complex photosystem II
(107-15 + 107-0) vs (90-15 + 90-0)	ESR42597	Ciclev10012370m	2.112	0.039	16218093		light harvesting complex photosystem II

Table S8. List of DEGs in *LCl-6* coded by transcript (mRNA), using the *P. trifoliata* genome as reference, per comparison. Information on the *C. clementina* (Ccl) orthologue, gene physical position (start), fold change expression (logFC), adjusted *p* value, and annotation, when known, is provided for each entry.

Comparison	mRNA	Ccl	logFC	padj	Start	Candidate	Anotation
(107-15 + 107-0) vs (90-15 + 90-0)	Ptrif.0006s0373.1	Ciclev10013664m	4.954	0.031	7953229		
(107-15 + 107-0) vs (90-15 + 90-0)	Ptrif.0006s0379.1	Ciclev10012754m	-1.081	0.069	8195685		TCP family transcription factor
(107-15 + 107-0) vs (90-15 + 90-0)	Ptrif.0006s0399.1		-6.102	0.006	8509586		DNAse I-like superfamily protein
107-15 vs 90-15	Ptrif.0006s0399.1		-4.188	0.023	8509586		DNAse I-like superfamily protein
(107-15 + 107-0) vs (90-15 + 90-0)	Ptrif.0006s2324.1	Ciclev10012331m	-1.404	0.022	8518847		RNA-binding (RRM/RBD/RNP motifs) family protein
(107-15 + 107-0) vs (90-15 + 90-0)	Ptrif.0006s0418.2	Ciclev10012379m	1.565	0.085	8821986	PIP2-1	
(107-15 + 107-0) vs (90-15 + 90-0)	Ptrif.0006s0421.1	Ciclev10011116m	2.263	0.055	8860889		CRINKLY4 related 1
(107-15 + 107-0) vs (90-15 + 90-0)	Ptrif.0006s0422.1	Ciclev10013919m	-1.828	0.007	8869539		
(107-15 + 107-0) vs (90-15 + 90-0)	Ptrif.0006s0428.1	Ciclev10012588m	3.412	0.055	8990818		FASCICLIN-like arabinogalactan-protein 11
(107-15 + 107-0) vs (90-15 + 90-0)	Ptrif.0006s0466.1	Ciclev10012036m	3.357	0.085	9519455		Nucleotide-diphospho-sugar transferases superfamily protein
(107-15 + 107-0) vs (90-15 + 90-0)	Ptrif.0006s0469.1	Ciclev10011643m	2.592	0.035	9555113		IQ-domain 2
(107-15 + 107-0) vs (90-15 + 90-0)	Ptrif.0006s0480.1	Ciclev10013573m	2.841	0.086	9682256		minichromosome maintenance 8
(107-15 + 107-0) vs (90-15 + 90-0)	Ptrif.0006s0504.1	Ciclev10011660m	0.862	0.091	10007630		WRKY family transcription factor family protein
(107-15 + 107-0) vs (90-15 + 90-0)	Ptrif.0006s0507.1	Ciclev10032010m	1.147	0.042	10041155		F-box family protein
(107-15 + 107-0) vs (90-15 + 90-0)	Ptrif.0006s0512.1	Ciclev10012631m	-1.189	0.034	10206685		Glutathione S-transferase family protein
(107-15 + 107-0) vs (90-15 + 90-0)	Ptrif.0006s0518.1	Ciclev10012410m	3.915	0.041	10262904		RING/U-box superfamily protein
(107-15 + 107-0) vs (90-15 + 90-0)	Ptrif.0006s0530.1	Ciclev10012844m	-2.153	0.034	10344501		(1 of 1) PF03692 - Putative zinc- or iron-chelating domain (CxxCxxCC)
(107-15 + 107-0) vs (90-15 + 90-0)	Ptrif.0006s0536.1	Ciclev10011291m	3.455	0.006	10455554		Fatty acid hydroxylase superfamily
107-15 vs 90-15	Ptrif.0006s0536.1	Ciclev10011291m	2.036	0.067	10455554		Fatty acid hydroxylase superfamily
(107-15 + 107-0) vs (90-15 + 90-0)	Ptrif.0006s0537.1	Ciclev10011291m	7.442	0.007	10467974		Fatty acid hydroxylase superfamily
(107-15 + 107-0) vs (90-15 + 90-0)	Ptrif.0006s0539.1	Ciclev10013485m	3.748	0.070	10505139		
(107-15 + 107-0) vs (90-15 + 90-0)	Ptrif.0006s0540.2	Ciclev10011167m	1.624	0.036	10512840	ABCG	
(107-15 + 107-0) vs (90-15 + 90-0)	Ptrif.0006s0541.1	Ciclev10011147m	2.104	0.057	10535146	ABCG	ABC-2 type transporter family protein
(107-15 + 107-0) vs (90-15 + 90-0)	Ptrif.0006s2342.1	Ciclev10011715m	-2.711	0.003	10578628		HXXXD-type acyl-transferase family protein
107-15 vs 90-15	Ptrif.0006s2342.1	Ciclev10011715m	-1.442	0.043	10578628		HXXXD-type acyl-transferase family protein

(107-15 + 107-0) vs (90-15 + 90-0)	Ptrif.0006s0553.1	Ciclev10013809m	-3.586	0.088	10706645		
(107-15 + 107-0) vs (90-15 + 90-0)	Ptrif.0006s0554.1	Ciclev10011951m	-1.260	0.057	10712358		
(107-15 + 107-0) vs (90-15 + 90-0)	Ptrif.0006s0567.2	Ciclev10012060m	-1.160	0.010	10892531		Tetratricopeptide repeat (TPR)-like superfamily protein
(107-15 + 107-0) vs (90-15 + 90-0)	Ptrif.0006s0572.2	Ciclev10010956m	2.907	0.078	10919502		ATP binding microtubule motor family protein
(107-15 + 107-0) vs (90-15 + 90-0)	Ptrif.0006s0579.1	Ciclev10012723m	-1.099	0.098	11003559		pyrophosphorylase 4
(107-15 + 107-0) vs (90-15 + 90-0)	Ptrif.0006s0593.1	Ciclev10012475m	-0.897	0.052	11128891		
(107-15 + 107-0) vs (90-15 + 90-0)	Ptrif.0006s0601.1	Ciclev10011060m	1.357	0.090	11185093		cation/H+ exchanger 20
(107-15 + 107-0) vs (90-15 + 90-0)	Ptrif.0006s2350.1	Ciclev10014295m	-7.338	0.006	11341441		
(107-15 + 107-0) vs (90-15 + 90-0)	Ptrif.0006s0610.1	Ciclev10011745m	1.769	0.003	11359669	MFS	Major facilitator superfamily protein
107-15 vs 90-15	Ptrif.0006s0610.1	Ciclev10011745m	1.071	0.023	11359669	MFS	Major facilitator superfamily protein
(107-15 + 107-0) vs (90-15 + 90-0)	Ptrif.0006s0626.1	Ciclev10013628m	1.965	0.092	11522906		Transducin/WD40 repeat-like superfamily protein
(107-15 + 107-0) vs (90-15 + 90-0)	Ptrif.0006s0635.1	Ciclev10013810m	-1.701	0.065	11632478		Protein of unknown function (DUF3741)
(107-15 + 107-0) vs (90-15 + 90-0)	Ptrif.0006s0649.2	Ciclev10012089m	-1.792	0.003	11913993		myb-like HTH transcriptional regulator family protein
107-0 vs 90-0	Ptrif.0006s0649.2	Ciclev10012089m	-1.093	0.072	11913993		myb-like HTH transcriptional regulator family protein
(107-15 + 107-0) vs (90-15 + 90-0)	Ptrif.0006s0659.1	Ciclev10012381m	-1.750	0.005	12037889		(1 of 1) PF01809 - Haemolytic domain (Haemolytic)
107-15 vs 90-15	Ptrif.0006s0659.1	Ciclev10012381m	-1.094	0.043	12037889		(1 of 1) PF01809 - Haemolytic domain (Haemolytic)
(107-15 + 107-0) vs (90-15 + 90-0)	Ptrif.0006s0678.1	Ciclev10011334m	1.491	0.088	12169108		Pathogenesis-related thaumatin superfamily protein
(107-15 + 107-0) vs (90-15 + 90-0)	Ptrif.0006s0687.1	Ciclev10013799m	1.221	0.057	12217045		Concanavalin A-like lectin protein kinase family protein
(107-15 + 107-0) vs (90-15 + 90-0)	Ptrif.0006s0690.1	Ciclev10011508m	2.925	0.049	12242385		Protein of unknown function (DUF604)
(107-15 + 107-0) vs (90-15 + 90-0)	Ptrif.0006s0695.1	Ciclev10012433m	-1.787	0.088	12301482		
(107-15 + 107-0) vs (90-15 + 90-0)	Ptrif.0006s0696.2	Ciclev10013780m	-6.081	0.017	12305174		
107-15 vs 90-15	Ptrif.0006s0696.2	Ciclev10013780m	-4.413	0.067	12305174		
(107-15 + 107-0) vs (90-15 + 90-0)	Ptrif.0006s0697.2	Ciclev10004349m	1.725	0.019	12310413		Homeodomain-like superfamily protein
(107-15 + 107-0) vs (90-15 + 90-0)	Ptrif.0006s2456.1	Ciclev10012494m	-2.700	0.029	12319292		
(107-15 + 107-0) vs (90-15 + 90-0)	Ptrif.0006s0706.1	Ciclev10011164m	1.628	0.027	12383996		
(107-15 + 107-0) vs (90-15 + 90-0)	Ptrif.0006s0708.1	Ciclev10013212m	3.453	0.011	12405094		(1 of 2) PTHR34670:SF3 - EXPRESSED PROTEIN
(107-15 + 107-0) vs (90-15 + 90-0)	Ptrif.0006s2359.1	Ciclev10012040m	-1.562	0.006	12444871		
107-0 vs 90-0	Ptrif.0006s2359.1	Ciclev10012040m	-1.106	0.072	12444871		

(107-15 + 107-0) vs (90-15 + 90-0)	Ptrif.0006s0715.1	Ciclev10012242m	2.169	0.007	12460159		NAD(P)-linked oxidoreductase superfamily protein
107-15 vs 90-15	Ptrif.0006s0715.1	Ciclev10012242m	1.429	0.066	12460159		NAD(P)-linked oxidoreductase superfamily protein
(107-15 + 107-0) vs (90-15 + 90-0)	Ptrif.0006s0740.1	Ciclev10011268m	0.816	0.070	12688857		POX (plant homeobox) family protein
(107-15 + 107-0) vs (90-15 + 90-0)	Ptrif.0006s2462.1	Ciclev10011341m	-1.353	0.012	12728591	NPF5.12	Major facilitator superfamily protein
(107-15 + 107-0) vs (90-15 + 90-0)	Ptrif.0006s0751.1	Ciclev10013132m	4.102	0.006	12765525		Bifunctional inhibitor/lipid-transfer protein
107-15 vs 90-15	Ptrif.0006s0751.1	Ciclev10013132m	2.448	0.066	12765525		Bifunctional inhibitor/lipid-transfer protein
(107-15 + 107-0) vs (90-15 + 90-0)	Ptrif.0006s0769.1	Ciclev10012250m	-1.248	0.034	12880735		Arabidopsis Inositol phosphorylceramide synthase 1
(107-15 + 107-0) vs (90-15 + 90-0)	Ptrif.0006s0774.1	Ciclev10011384m	1.427	0.065	12916342		laccase 7
(107-15 + 107-0) vs (90-15 + 90-0)	Ptrif.0006s0778.2	Ciclev10011375m	2.998	0.013	12956964		laccase 7
(107-15 + 107-0) vs (90-15 + 90-0)	Ptrif.0006s0785.2	Ciclev10011279m	2.938	0.010	12998591		O-fucosyltransferase family protein
(107-15 + 107-0) vs (90-15 + 90-0)	Ptrif.0006s0790.1	Ciclev10011563m	-0.675	0.088	13035167		RING/U-box superfamily protein
(107-15 + 107-0) vs (90-15 + 90-0)	Ptrif.0006s0799.1	Ciclev10013019m	5.429	0.030	13096096		
(107-15 + 107-0) vs (90-15 + 90-0)	Ptrif.0006s0802.1	Ciclev10013166m	-1.596	0.098	13121317		Protein of unknown function (DUF962)
(107-15 + 107-0) vs (90-15 + 90-0)	Ptrif.0006s0809.1	Ciclev10011239m	1.756	0.065	13234551		Protein of unknown function (DUF740)
(107-15 + 107-0) vs (90-15 + 90-0)	Ptrif.0006s0814.5	Ciclev10011381m	2.037	0.007	13277480	NPF8.1	peptide transporter 1
(107-15 + 107-0) vs (90-15 + 90-0)	Ptrif.0006s0815.1	Ciclev10013821m	6.161	0.086	13284465	NPF8.2	
(107-15 + 107-0) vs (90-15 + 90-0)	Ptrif.0006s0816.1	Ciclev10011400m	3.413	0.079	13290531		Laccase/Diphenol oxidase family protein
(107-15 + 107-0) vs (90-15 + 90-0)	Ptrif.0006s0817.1	Ciclev10012285m	1.799	0.010	13300960		Duplicated homeodomain-like superfamily protein
(107-15 + 107-0) vs (90-15 + 90-0)	Ptrif.0006s0823.1	Ciclev10013337m	6.937	0.006	13354423	NPF5.9	proton-dependent oligopeptide transport (POT) family protein
107-15 vs 90-15	Ptrif.0006s0823.1	Ciclev10013337m	3.530	0.078	13354423	NPF5.9	proton-dependent oligopeptide transport (POT) family protein
(107-15 + 107-0) vs (90-15 + 90-0)	Ptrif.0006s0827.1	Ciclev10011536m	2.125	0.052	13385377		glycerol-3-phosphate acyltransferase 6
(107-15 + 107-0) vs (90-15 + 90-0)	Ptrif.0006s0838.2	Ciclev10013652m	-1.669	0.014	13506477		
(107-15 + 107-0) vs (90-15 + 90-0)	Ptrif.0006s0841.2	Ciclev10013439m	-3.980	0.010	13552749		
(107-15 + 107-0) vs (90-15 + 90-0)	Ptrif.0006s0848.4	Ciclev10011409m	-1.048	0.040	13610864		Carbohydrate-binding-like fold
(107-15 + 107-0) vs (90-15 + 90-0)	Ptrif.0006s0854.1	Ciclev10012370m	2.539	0.017	13655960		light harvesting complex photosystem II
(107-15 + 107-0) vs (90-15 + 90-0)	Ptrif.0006s0866.1	Ciclev10012918m	-2.477	0.006	13765578		
(107-15 + 107-0) vs (90-15 + 90-0)	Ptrif.0006s0901.1	Ciclev10011366m	1.107	0.030	14033606		DNAJ heat shock N-terminal domain-containing protein
(107-15 + 107-0) vs (90-15 + 90-0)	Ptrif.0006s0902.2	Ciclev10011094m	1.866	0.058	14040413		Pentatricopeptide repeat (PPR) superfamily protein

(107-15 + 107-0) vs (90-15 + 90-0)	Ptrif.0006s2379.1	Ciclev10014534m	0.938	0.088	14050230	
(107-15 + 107-0) vs (90-15 + 90-0)	Ptrif.0006s0918.2	Ciclev10011595m	1.046	0.098	14141796	ammonium transporter 2
(107-15 + 107-0) vs (90-15 + 90-0)	Ptrif.0006s0923.2	Ciclev10010996m	0.811	0.092	14177509	tetratricopeptide repeat (TPR)-containing protein
(107-15 + 107-0) vs (90-15 + 90-0)	Ptrif.0006s0932.1	Ciclev10013559m	5.945	0.031	14231262	
(107-15 + 107-0) vs (90-15 + 90-0)	Ptrif.0006s2383.1	Ciclev10011480m	-0.813	0.029	14249683	(1 of 4) PF14237 - Domain of unknown function (DUF4339) (DUF4339)
(107-15 + 107-0) vs (90-15 + 90-0)	Ptrif.0006s0939.1	Ciclev10012499m	-0.864	0.022	14254882	Ribosomal protein S4 (RPS4A) family protein
(107-15 + 107-0) vs (90-15 + 90-0)	Ptrif.0006s0947.1	Ciclev10013878m	-1.071	0.055	14296840	TRICHOME BIREFRINGENCE-LIKE 35
(107-15 + 107-0) vs (90-15 + 90-0)	Ptrif.0006s0968.1	Ciclev10011564m	-2.393	0.022	14447210	iron regulated 1
(107-15 + 107-0) vs (90-15 + 90-0)	Ptrif.0006s0973.1	Ciclev10012298m	2.233	0.035	14482241	NAD(P)-binding Rossmann-fold superfamily protein
(107-15 + 107-0) vs (90-15 + 90-0)	Ptrif.0006s0982.1	Ciclev10012456m	-2.060	0.015	14529686	Protein of unknown function (DUF567)
(107-15 + 107-0) vs (90-15 + 90-0)	Ptrif.0006s2386.1	Ciclev10012606m	-1.297	0.086	14639604	
(107-15 + 107-0) vs (90-15 + 90-0)	Ptrif.0006s0996.1	Ciclev10026054m	3.022	0.012	14670806	
(107-15 + 107-0) vs (90-15 + 90-0)	Ptrif.0006s1003.1	Ciclev10011350m	3.270	0.052	14719145	
(107-15 + 107-0) vs (90-15 + 90-0)	Ptrif.0006s1004.2	Ciclev10011771m	-1.476	0.006	14734192	serine/threonine protein kinase 1
(107-15 + 107-0) vs (90-15 + 90-0)	Ptrif.0006s1005.1	Ciclev10011217m	1.848	0.023	14749634	ARM repeat superfamily protein
(107-15 + 107-0) vs (90-15 + 90-0)	Ptrif.0006s1008.1	Ciclev10012094m	3.546	0.070	14765782	2-oxoglutarate (2OG) and Fe(II)-dependent oxygenase protein
(107-15 + 107-0) vs (90-15 + 90-0)	Ptrif.0006s1017.2	Ciclev10013804m	3.202	0.012	14822836	
(107-15 + 107-0) vs (90-15 + 90-0)	Ptrif.0006s1035.2	Ciclev10010994m	1.881	0.052	14933272	Leucine-rich receptor-like protein kinase family protein

Table S9. Primer sequences for mapping candidate genes and genetic positions of the corresponding SCAR markers.

Gene	Forward Primer	Reverse Primer	Genetic Position 4c (cM)	Genetic Position R4c (cM)
PIP2.1	CGGTAAGCWGCARGGAATCA	KCCCAAATGAGTGTCCACAA	62.906	29.325
ABCG	CTTAGGGGCGACATGTTTGG	AAGAAGAGAAGCAAGCAAATGAA	33.8	19.775
MSF	GGAGCTATTGCTCAGGTGGT	TCCTGTTCCCAGAAGCAATC	17.437	14.458
NPF5.12	CACGCACCAATATCCAAAGA	TTTCGATGCCATCAATTCTG	11.102	8.344
NPF8.1	CTTAGGGGCGACATGTTTGG	AAGAAGAGAAGCAAGCAAATGAA	10.007	.
NPF5.9	GGCTCAAATTAAGCGTGTCC	TATTACCGAACAAGACGCTTAGGC	3.234	3.177

Table S10. Sequences of SCAR markers. Comparisons with *C. clementina* orthologous genes using https://phytozome-next.jgi.doe.gov/blast-search.

-MSF from Flying Dragon (Ciclev10011745m.g: E-value=0; % identity= 98; Alignment length= 501)

GCAATGTGAGCTTGAATGTATTAATATTTTTCCATCTCCTTGATTCACTGTCTTCC-TATAGGGTAACTAGTGGAGTACTTGGCACTCTATATCCACTTATCATGGCAGCTTTGTTGGGCATTGGTGATGGAGTGCTCAATACACAGCTTAGTGCTTTGCTTGGGATACTCTTTAAGCACGATACGGTAACAGCTAATCCTACTTTAATAAGACAAATATTTGTTTGGTAGGTGAC-TATGAAATTTCCTTTTAGTCAGTCAGTCTACCTCATATTCTTCAGGAACACCCAAGGATACAATGGCAATGATGAAACTAGTCATATCAGCAATATTCTGCATCAAACTTGCTTATTTTATTTTACCTGGCAAATCATATTC-TATTAGGTTCATATTTTTCCATATCTAATCATAAAGGAAAAGAAAATGTAAAAAAATGAAAATTCCACCTACTGTATTAGATGGTTGCTTTGTATATAGGTCTAGAATTCTTGCAAGATGGAGAG

-MSF from 22-7 (Ciclev10011745m.g: E-value=0; % identity= 99; Alignment length= 790)

GAGAGATTACAAATTAAATAAAAAGAAAACAATGACCTAATTTAGGCTTCTTTTACT-AATCAGCATAAAAAGAATCATGTATAAACTTATCCGTAATCTGTTAAATGAGGACGCCAACCATAGTAGCTAATTGGCTAATTTATAACACAAAAGAAAAGTCTCTCCTCCATCTTGCAAGAATTCTAGACCTATATACAAAGCAACCATCTAATACAGTACGTG-GAATTTTCATTTTTTTACATTTTCTTTTCCTTTATGATTGGATATGGAAAAATATGAACCTAATAGAATATGATTTGCCAGGTAAAATAAAATAAGCAAGTTTGATGCAGAATATTGCTGATATGACTAGTTTCATCATTGCCATCGTATCCTTGGGTGTTCCTGAAGAT-TATGAGGTAGACTGACTGACTAAAAGGAAATTCATATTCATAGTCACCTACCAAACAAGTATTTGTCTTATTAAAGTTGGATTAGCTGTTACCGTATCGTGCTTAAAGAGTATCCCAA

GCAAAGCACTAAGCTGTGTATTGAGCACTCCATCACCAATGCCCAACAAAGCTGCCATGA-
TAAGTGGATATAGAGTGCCAAGTACTCCACTAGTTACCCTATAGGAAGACAGTGAATCAAGGAGATGGAAAAATATTAATACATTCAAGCTCACACTACTACAAAACAACCTTCAA
GTTCTGAAAACTTCAATGTAACAGGAGTACACAACACAGTGTAACAGGAGTACACAACACTAACCTGTAATTTATCAGAATCCAGAGAAATACAACCAC

-NPF5.9 from 22-7 (Ciclev10013337m.g: E-value=0; % identity= 99; Alignment length= 631)

GTGCAGTGTTACCGAAGTTGAAGAAACAAAATGTCTTGTACATTTGATTCCAATTT-
GTTTCACTTTCATCATACCAGGAGTAGTTTCCTCAATTGGAAACACTTTCTTTCTTGAGCAAGCAGAAGTTATAAACGGAAAAGATGGAGGAAATTTTAGTTTCGGTCCTCTATTGCT
CCTACTGTTGTATTACATCTCGAAAGACCGTTTCACGGCCTTCTACGAAGGATTTGTT-
GGACATCTCACTTCAGTAGGATGGAACGCCGTAGCTCATGTCGTTGGATTTGCAATGTCAATGATTCTATCAATACTATGCTGTGTAACAGCAGCAATAGTCGAGACTCGGAGGTT
GGATGTTGTTAGAAGCCATGGTTTAATTGACAAGCCTGATGAAAAAATCCCCATGAGCTT-
GTTTTGGCTGCTGCCACAGTTTCTACTTCTCGGAGCCGCTGATGGGTTCTATGAAAAAGGCATTGATATGTTCTTCAGCGATCAGTTTCCTCGCATGATTTTCAGATATCTGAAGTAC
ATTTTACTATGGGAGTAGGAATTATTGGCAGTACCCTGTCCGTTTACTATGCGGGAATCATCAGTGAAAGGATCGGGAAACATAATTGGTTTCAGGAC

-NPF5.9 from Flying Dragon (Ciclev10013337m.g: E-value=0; % identity= 99; Alignment length= 629)

GACGATTCCGGCATAGTAAACGGACAGGGTACTGCCAATAATTCCTACTCCCATAGTAG-
CAACAAAAATGTACTTCAGATATCTGAAAATCATGCGAGGAAATTGATCGCTGAAGAACATATCAATGCCTTTTTCATAGAACCCATCAGCGGCTCCGAGAAGTAGAAACTGTGGC
AGCAGCCAAAACATGCTCATGGGGATTTTTTCATCAGGCTTGTCAATTAAAC-
CATGGCTTCTAACAACATCCAACCTCCGAGTCTCGACTATTGCTGCTGTTACACAGCATAGTATTGATAGAATCATTGACATTGCAAATCCAACGACATGAGCTACGGCGTTCCATCC
TACTGAAGTGAGATGTCCAACAAATCCTTCGTAGAAGGCCGTGAAACGGTCTTTCGA-
GATGTAATAGAGCAGTAGGAGCAATAGAGGACCGAAACTAAAATTTCCTCCATCTTTTCCGTTTATAACTTCTGCTTGCTCAAGAAAGAAAGTGTTTCCAATTGAGGAAACTACTCC
TGGTATGATGAAAGTGAAACAAATTGGAATCAAATGTACAAGACATTTTGTTTCTTCAACTTCGGTAACACTGCACAATCTCCATCTGTTTCTTTCTTGTTGCCTAAGCG

- NPF5.9 from 90, allele c (Ciclev10013337m.g: E-value= 6.47e-92; % identity= 98; Alignment length= 199)

ATCCTTTCCTGACGATTCCGGCATAGTAAACGGACAGGGTACTGCCAATAATTCC-
TACTCCCATAGTAGCAACAAAAATGTACTTCAGATATCTGAAAATCATGCGAGGAAATTGATCGCTGAAGAACATATCAATGCCTTTTTCATAGAACCCATCAGCGGCTCCGAGAAG
TAGAAACTGTGGCAGCAGCCAAAACATG

- NPF5.9 from Cleopatra, allele b (Ciclev10013337m.g: E-value= 1.36e-100; % identity= 100; Alignment length= 203)

CGCTGATGGGTTCTATGAAAAAGGCATTGATATGTTCTTCAGCGATCAGTTTCCTCG-
CATGATTTTCAGATATCTGAAGTACATTTTTGTTGCTACTATGGGAGTAGGAATTATTGGCAGTACCCTGTCCGTTTACTATGCGGGAATCATCAGTGAAAGGATCGGGAAACATAA
TTGGTTTCAGGACACGCTTAATTTGAGCC

-ABCG from 22-7 (Ciclev10013485m.g: E-value=2.31e-101; % identity= 100; Alignment length= 205)

GTTTAACAGTATGGACTACTTCCAAAGATATTAAAAAAATTCAAAAAAGGAATGTG-
GACATGATTTCTTTTGCTATTATAAGATTCGTTTTAAAAGTTAAATTCAAAGCCCACCAAGTTATATATGCATTGCATTTCCATTCCGAATTTGGATCCAAACATATTAGCATTGTCATT
ATGGAGGCACCAAACATGTCGCCCCTAAGGACCTTCTTAGAAATATCGGCTCCCACATCCG-
CATCCTCAAGAGCCTTCTTGCCAAAAACAATGCTGAACCTCAAACAATCATCAATTCTGCGATCATTCTTGCCATCAATATAACCCTCTGCACTAATTCCCCGGATCTGGTCGCCGAA
GCGGGTCGGGAACTTACTAGCATCGAACCGGTCAATC

-NPF5.12 from 22-7 (Ciclev10011341m.g: E-value=0; % identity= 96; Alignment length= 453)

GATGCTAGCTCCTCTGCGCATGGATAGCTATCTCTCACGTATA-
GAATTAATCCTTTTTCTTTTTCTTTTTCTTTTTTTACCTTCTTTTTTCCTTCCTTTTCTTAATTCTCTTCAAGACAAGATTATATATATATAGACACAAACACACACACACACAATTTAATTA
TTTGAATTTTTAAACTAG-
TGATCCTCATATTCTCAAGTAACATCATGCCTCAAAAAAAAAATCATAAATGTGCCATTCCTATTATCTTGGGAGTCCTTCAAGATGTCTGTATTTTTATAAAGTTTAAACTCACACCA
CTATATAGCAGTATAAAAGTCTTTACAACATTATAAATTAAAA-
GCATGTAAAAAAATCCTTCATTATACTGTAAAGGTGCCAGTTTACAACTTATAAAGTGTGAATGTCTGCACCAGATAATAACTATATCTTTG

-NPF5.12 from Fd (Ciclev10011341m.g: E-value=1.84e-140; % identity= 83; Alignment length= 447)

ACTTTACAAGTTGTAAACTCTCACCTTTACAG-
TATAATGAAGGATCTTTTACATGCTTTTAATTTATAATGTTGTAAAGACTTTTATCCTGCTATATAGTGGTGTGAGTTTAAACTTTATAAAAATACAGACATCTTGAAGGACTCCCAAG
ATAATAGGAATGGCACATTTATGATGTTACTTGAGTATATGAGGATCACTAGTTTAAAGAT-
TCAAATAATTAAATTGTGTGTGTGTCTATATATATATAATCTTGTCTTGAAGAGAATTAAGAAAAGGATGGAAAAAAGAAAAAGAAAAAGGATTAATTCTATACGTGAGACATAGC
TATCCATGCGCAGAGGAGCTAGCATTCAGAAAAACGAAGAGAAGCAGCAGAATT

- PIP2.1 from Fd [genomic sequence near Ciclev10012375m.g (scaffold_6:10422129..10422358), at scaffold_6:10422321..10422400: E-value= 1.07e-32; % identity= 99; Alignment length= 80)

TGATCAGGGAATGCATTGCCAAATTATTGTAATGTTAAGAAAAGAAAGTGCCCCGTCCCATTTGTGGACACTCATTTGGGC

- PIP2.1 from 90, allele b, [genomic sequence near Ciclev10012375m.g (scaffold_6:10419232..10420904), at scaffold_6:10422321..10422400: E-value= 2.02e-89; % identity= 96; Alignment length= 210)

GAGTTGCTGGAAGCGTGCAAGTACGGGTTTGCCGGGTAAGATTGATTTGAAATACGAAAA-
TATTTGGAAGTGGATTTAAGTGATCAGGGAATGCATTGCCAAATTATTGTAATGTTAAGAAAAGAAAGTGCCCCGTCCCATTTGTGGACACTCATTTGGGCCATTGTGGACACGCC
GTTTGGGAATAATTTGGCAATGCATCCCTGATCACTTAAATCCACTTCCAAATATATTCG-
TATTTCAAATCAATCTTACCCGGCAAACCCGTACTTGCACGCTTCCGCAACTTGTTTTCAACCACTACCTGCATAGTTGCGTGCACTCAATACATGCGTTGTTTCACTACTTGCATAGTT
GCATGCACTCAGTACATGCATTGTTTTGTCAAAAA

- PIP2.1 from 90, allele c, [genomic sequence near Ciclev10012375m.g (scaffold_6:10419232..10420904), at scaffold_6:10422219..10422409: E-value= 6.40e-70; % identity= 95; Alignment length= 173)

AGTGGTAGAAAACAAGTTGCTGGAAGCGTGCAATTACGGGTTGGGGTAAGATTGATTTGAAA-
TACGAAAATATTTGGAAGTGGATTTAAGTGATCAGGGAATGCATTGCTAAATTATTGTAATGTTAAGAAAAGAAAGTGCCCCGTCCCATTTGTGGACATCATTTGGGCATTTGGGC
ATTTCTTAACATTAAATAATTTAGCATGCATTCCCTGATCACTTAAATCCACTTCCAAA-
TATTTTCGTATTTCAAATCAATCTTACCCCAACCCGTAATTGCACGCTTCCGCAACTTGTTTTCTACCACTACTTGCATAGTTGCATGCACTCAGTTCAAGCGTTGTTTTGTCAAAAGCT

- PIP2.1 from 107, allele a, [genomic sequence near Ciclev10012375m.g (scaffold_6:10419232..10420904), at scaffold_6:10422160..10422411: E-value= 3.87e-112; % identity= 99; Alignment length= 230)

TGCTGCAACTATGCAAGTAGTGAAACAACGCATGTAATGAGTGCACGCAACTATGCAAGTAG-
TGGTTGAAAACAAGTTGCTGGAAGCGTGCAAGTACGGGTTTGACGGGTAAGATTGATTTGAAATACGAAAATATTTGGAAGTGGATTTAAGTGATCAGGGAATGCATTGCCGAAT
TATTGTAATGTTAAGAAAAGAAAGTGCCCCGTCCCATTTGTGGACATCCATTTGGGCCAAC-
GTTCACACTGGTTTGGGCAATAATTCGGCAATGCATTCCCTGATCACTTAAATCCACTTCCAAATATTTTCGTATTTCAAATCAATCTTACCCGTCAAACCCGTACTTGCACGCTTCCA
GCAACTTGTTTTCAACCACTACTTGCATAGTTGCGTGCACTCAATACATGCGTTGTTTCACTACTTGCATAGTTGCAAGCACTAAGTACATGCGTTGTTTTGTCAAAAAG

- PIP2.1 from 107, allele c, [genomic sequence near Ciclev10012375m.g (scaffold_6:10419232..10420904), at scaffold_6:10422125..10422386: E-value= 2.81e-67; % identity= 92; Alignment length= 185)

ATTGATTTGAAATACGAAAATATTTGGAAGTGGATTTAAGTGATCAGGGAATGCATT-
GCTAAATTATTGTAATGTTAAGAAAAGAAAGTGCCCCGTCCCATTTGTGGACCTCATTTGGGCCACTTTCATTTCTTAACATTAAATAATTTAGCAATGCATTCCCTGATCACTTAAAT
CCACTTCCAAATATTTTCGTATTTCAAATCAATCTTACCCCAACCCGTAATTGCAC-
GCTTCCGCAACTTGTTTTCTACCACTACTTGCATAGTTGCATGCACTCAGTTCATGCGTTGTTTTGTCAAAAAGT

- NPF8.1 from 22-7 (Ciclev10011381m.g: E-value= 1.98e-78; % identity= 99; Alignment length= 169)

TTTAAACCAATATAGCAAATATCGATACTCTAGATTAAAAATTTGATATTTAAAACTA-
TATGTACAAAGCATTTTCTTTTATCAATCACAAAAAAACTGAAACTCCGAAAGAAAACACACACATTGATCCACCTACCATTCCTTCTATCAATTTGCACGTCCAGTAGCC

- NPF8.1 from Cleopatra (Ciclev10011381m.g: E-value= 4.14e-74; % identity= 99; Alignment length= 159)

CATGTGTGTGTTTTCTTTCGGAGTTTCAGTTTTTTTGTGATTGATAAAAGAAAATGCTTT-
GTACATATAGTTTTAAATATCAAATTTTTAATCTAGAGTATCGATATTTGCTATATTGGTTTAAATTTTTGGGGTTCGATTTATGCTTGCAGTTCCCGTTTG
